# Random embedded calibrated statistical blind steganalysis using cross validated support vector machine and support vector machine with particle swarm optimization

**DOI:** 10.1038/s41598-023-29453-8

**Published:** 2023-02-09

**Authors:** Deepa D. Shankar, Adresya Suresh Azhakath

**Affiliations:** 1 Abu Dhabi University, Abu Dhabi, United Arab Emirates; 2 Department of Health Technology, Danmarks Teknikse Universitet, Copenhagen, Denmark

**Keywords:** Computer science, Statistics

## Abstract

The evolvement in digital media and information technology over the past decades have purveyed the internet to be an effectual medium for the exchange of data and communication. With the advent of technology, the data has become susceptible to mismanagement and exploitation. This led to the emergence of Internet Security frameworks like Information hiding and detection. Examples of domains of Information hiding and detection are Steganography and steganalysis respectively. This work focus on addressing possible security breaches using Internet security framework like Information hiding and techniques to identify the presence of a breach. The work involves the use of Blind steganalysis technique with the concept of Machine Learning incorporated into it. The work is done using the Joint Photographic Expert Group (JPEG) format because of its wide use for transmission over the Internet. Stego (embedded) images are created for evaluation by randomly embedding a text message into the image. The concept of calibration is used to retrieve an estimate of the cover (clean) image for analysis. The embedding is done with four different steganographic schemes in both spatial and transform domain namely LSB Matching and LSB Replacement, Pixel Value Differencing and F5. After the embedding of data with random percentages, the first order, the second order, the extended Discrete Cosine Transform (DCT) and Markov features are extracted for steganalysis.The above features are a combination of interblock and intra block dependencies. They had been considered in this paper to eliminate the drawback of each one of them, if considered separately. Dimensionality reduction is applied to the features using Principal Component Analysis (PCA). Block based technique had been used in the images for better accuracy of results. The technique of machine learning is added by using classifiers to differentiate the stego image from a cover image. A comparative study had been during with the classifier names Support Vector Machine and its evolutionary counterpart using Particle Swarm Optimization. The idea of cross validation had also been used in this work for better accuracy of results. Further parameters used in the process are the four different types of sampling namely linear, shuffled, stratified and automatic and the six different kernels used in classification specifically dot, multi-quadratic, epanechnikov, radial and ANOVA to identify what combination would yield a better result.

## Introduction

With the advent of technology and digitalization of services, several crucial data and personal data have been vulnerable to cyber-attacks. The bank transactions, communication between government organizations or personnel, national defense units etc. generate high amount of critical information which demand security systems to prevent loss or corruption of data. Steganography and Cryptography are two frequently used information security techniques for efficient and confidential communication. Several instances in real life use multiple methods for a resilient security system.

**Figure 1:**
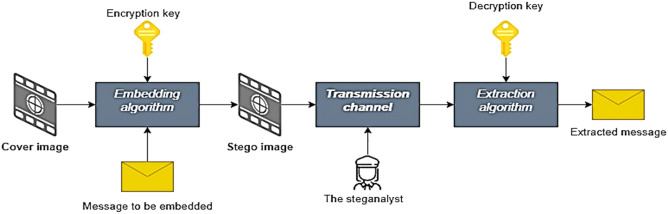
Block diagram of the steganographic process.

The confidential hiding of data for communication between a sender and a designated receiver is called steganography. This incognito data transfer can be done using copious types of multimedia formats like text, image, audio video and animation. The process of steganography consists of two algorithms-embedding and extraction. The embedding algorithm embeds or conceals the data into the medium that is used for the communication. In this paper, the medium used are JPEG images. JPEG is one of the most used formats as it is a standard image format used in scanners, photography, and other image processing tools.JPEG format is used in this paper due to ideal property of lossy compression which is necessary in critical information exchange. Hence, the images further to the embedding process will be called the stego image and it is transmitted using a transmission channel or the communication medium. Steganalysis which is a technique that intents to detect the presence of a hidden message is performed by a steganalyst at the communication channel. If the messages surpass the steganalyst, it reaches the receiver where the message is retrieved using the extraction algorithm. Like cryptography, the technique of steganography also uses a key which is shared only between the sender and receiver. The key is the most vital entity for both embedding and extraction algorithms^1^. The process is explained in Fig. 1. The image embedding in the paper is performed in two different domains-Spatial domain and Transform domain. The embedding in spatial domain is done right on the picture pixels whereas in the Transform domain the image is transformed using Discrete Cosine Transform to the coefficients. In the work, the steganographic algorithms used in the spatial domain are Least Significant Bit Matching (LSB M), Least Significant Bit Replacement (LSBR) and Pixel Value Differencing (PVD). F5 steganographic scheme is used in the transform domain.

Cryptography is a technique used for secret communication. In this technique the plaintext or the message to be sent is converted into cipher text through a process called encryption. To retrieve the message at the destination, decryption algorithm is used. An encryption and decryption key are used for both the processes respectively. The two types of cryptographic algorithm- asymmetric and symmetric, depend on the number of keys used in the cryptographic process. Symmetric algorithm is used when both the encryption and decryption stage used the same key to conceal and retrieve the message. Asymmetric algorithm is used if two different keys are used, each by the sender and receiver. In cryptography, the message is merely converted into cipher text, and it can render to the suspicion and increased data leak. However, the message in steganography remains benign and the presence itself is unknown. This is because the quality of a steganographic algorithm depends on its imperceptibility^2^. Due to this reason, many steganographic algorithms aims their best to hide the image while maintaining the imperceptibility. The steganographic algorithm can work both spatial and transform domain. In spatial domain techniques, the pixel values of an image are modified to hide the message. However, in the transform domain, the message to be hidden is embedded in the domain coefficients. Thus, such steganographic algorithms are resistant to geometric attacks and hard to identify using statistical steganalysis^3^. Hence making steganography a more efficient technique used for surreptitious communication as compared to cryptography.

Figure 1 explains the process of steganography which clearly illustrates the cover image, stego image, steganalyst and the keys.

In the recent years, the technique of steganography has been misused by bad actors. The news media in India had stated the possibility of steganographic communications in the 2006 attack of Bombing in Mumbai^4^. In 2013, Kaspersky which is a Russian security organization along with its partner, CrySys discovered the MiniDuke. The MiniDuke attackers used perceptive social engineering skills to decoy their targets by formatting PDF documents in a highly germane approach. The malware was downloaded to the system through the PDF files to find pre-made accounts for precise tweets for the retrieval of C2 or the command-and-control operators. Further to C2 location, the malware conceals itself as images to carry out cyberespionage actions^5^. The remote access Trojan, Berbomthum was reported by TrendMicro which would take commands from a twitter account which was created on 2017.The account used harmless looking malevolent memes which used steganography as a technique for communication between the malware and the programmers or attackers^6^.

Steganalysis is a method of identifying the steganographic scheme. The method is different from cryptanalysis as cryptanalysis emphases on recovery of the veiled message, whereas steganalysis focuses on the detecting the presence of the message. The basic types of steganalysis are the blind and targeted steganalysis. When the steganographic algorithm is formerly known by the steganalyst, it is called the targeted steganalysis. Blind steganalysis is when the algorithm is unknown to the analyst.

This aim of this research is to detect the presence of an embedded message in an image with a certain level of accuracy, and yet keeping a balance of the computational complexity and memory usage. This work would only be applicable for Joint Photographic Experts Group (JPEG) format with an image size of 256 × 256. The research is block based and hence the evaluation is done on 8 × 8 blocks. Text message is embedded into the image. The image before embedding is termed as cover image and the ones after the embedding is known as stego image. The message embedding capacity in the image is random. The selection of randomness is to check the accuracy of the classifier at different embedding percentages. This paper uses statistical blind steganalysis for the research. A combination of interblock and intrablock features are considered to eliminate the drawbacks of each one considered separately. The features extracted in the work include the first order, second order and the Markov features so that the minute changes and details can be noted for the binary classification of stego and cover image. The technique of calibration is also used to understand an approximation of the cover image from the stego image. This process is beneficial for blind steganalysis as the cover image is not known previously. Dimensionality Reduction using Principal Component Analysis (PCA) and tenfold cross validation techniques is also used in this paper before the features are fed into the classifiers. Standard datasets are used as both training and test data since these datasets for research are “clean” without noise or other interferences. The research uses Support Vector Machine (SVM) and its evolutionary variant Support Vector Machine-Particle Swarm Optimization (SVM-PSO) to have an estimation of the classification result under the same conditions.

The contribution of the proposed work is as follows:

- The block- based steganalysis approach in incorporated in this paper. The approach analyzes the image in terms of 8X8 blocks. This would improve the accuracy of the analysis since the image is analyzed in terms of blocks rather than one single frame. This would also enhance the real time scenario since the accuracy will be better even with a single test image.
- The format of image used in this paper is JPEG. It is because of the wide real time use of the format in the internet transmission due to its high compression ratio.
- The research uses two standard databases- INRIA holiday datasets as training data and UCID dataset as test data.
- The message embedding is done using text.
- Random percentage of embedding is used in this paper for the analysis.
- Four different steganographic algorithms namely LSB Replacement, LSB Matching, Pixel Value Differencing in the spatial domain and F5 in the transform domain is used for the random text embedding.
- After the random embedding, the next step is to extract the relevant features from the image. The features extracted are the first order, the second order, extended DCT and Markovian features. These features are a combination of inter-block features and intra-block features. This combination is used to eliminate the dependencies of both taken one at a time.
- The concept of cross validation of features are incorporated after extraction, for an improved estimation and prediction.
- Supervisory machine learning algorithm namely Support Vector Machine along with its evolutionary optimization counterpart known as Particle Swarm Optimization is used for a comparative study of the above extracted features during the classification.
- Six diverse kernel functions namely polynomial, dot, radial, epanechnikov, quadratic and ANOVA are used for classification in both classifiers. During the classification, four sampling techniques namely linear, shuffle, stratified and automatic is being used.
- To conclude, the classification rates are measured for accuracy and a comparative study is done for each scenario.

The organization of this paper is as follows. The next section "Related work" discuss about the related work, followed by the section “Problem statement”. “Dastset” gives an overview on the dataset. Section “Methodology” explain the methodology with "Preprocessing", "Block-Based Image Steganalysis", JPEG basics", "Steganographic algorithms", “Features for Extraction”, "Calibration", “Classification”, “Cross-validation”, “Classification kernels”, “Sampling”. “Results” gives the results of the above classification combinations. “Conclusion” concludes the inference and the last section details the ’Future scope” of the paper.

## Related work

Javad et al.^7^ proposed an innovative quantum model that consisted of sections-steganography and steganalysis for audio signals. The embedding operation in steganography is carried out within the LSFQ or the Least Significant Fractional Qubit. This is done to increase the Signal to Noise Ratio and to minimize the effects of the embedding process. The Steganalysis section consists of an analyzer to differentiate the cover and stego audio signals using statistical feature extraction. The process uses quantum circuits for the implementation of K-nearest neighbor algorithm and the Hamming Distance Criterion. The proposed algorithm yields high detection accuracy of over 80% and proves to be more efficient and secure as compared to several other methods proposed previously.

The related work on steganalysis system stated that the block based system put forth better results than a frame based system^8^, SeongHo Cho et al. had also proposed a steganalysis system using block based system and multiclassifier^9^. The advantages cited in the research had prompted to use the same concept in the research.

Ahn et al.^10^ proposed a method based on Local-source Enhanced Residual Network (LSER) along with end-to-end learning which demonstrates effective results as compared to steganalysis domains of JPEG and spatial. The residual blocks in the LSER are not normalized and a local-source skip connection is supplemented in the further step to improved feature representation.

Various steganographic techniques in both spatial and transform domain had been used in the research. A comparative study in spatial and transform embedding with less embedding had been done by Adresya et al.^11,12^ where there are good results shown for different kernels. A novel CNN based Text steganalysis was proposed by Wen et al.^13^ to understand the feature representations and complex dependencies from texts. The semantic and syntax features of words are extracted initially by the word embedding layer. The sentence features are learned using rectangular convolution kernels or varying sizes. A decision strategy is also introduced for detection of long texts. A novel technique for text steganalysis was proposed by Bao et al.^14^ using Attentional Long-Short Term Memory (LSTM)-Convolutional Neural Networks (CNN). The presented technique uses exploitation of the semantic features in texts and uses the LSTM-CNN model for acquisition of the contextual information of steganographic texts. The softmax layer is used to distinguish the cover text from the stegotext. You et al.^15^ proposed a method for image steganalysis based on a Siamese CNN architecture which comprises of two symmetrical subnets of shared parameter and three different stages from preprocessing followed by feature extraction to classification. The validation set contains image datasets of varying sizes. Su et al.^16^ proposed an advanced CNN architecture where the Gauss partial derivative filters are combined with ResNeXt. The GPD filters, generated residual images and were efficient in capturing the textural noise and disturbance in the edge regions. The ResNeXt was used to generate the image features by exploiting the residual images. The method was proven to have better performance results as compared to the CNN models-SCA-GFR and J-Xu-Net. Singh et al.^17^, in his work, introduced a steganalysis scheme using learned denoising kernels to facilitate increased precision of noise residual. The scheme consists for two steps- Initially a denoising CNN is trained followed by the used of the denoising CNN model to extract steganographic noise residual to train the classifier in distinguishing the cover images from stego images. Wu et al.^18^ presents an enhanced calibration-based universal JPEG steganalysis. The features generated in the process is based on the difference calculated between the test input/image and the equivalent macroscopic or microscopic calibrated input/image. The method uses Block Discrete Cosine Transform Coefficients and Markov empirical transition matrices.

Jia et al.^19^ proposed a method to solve the mismatch problems caused by the variations in statistical distribution between training and test sets causing reduced performance of algorithms in steganography. The algorithm proposed is a transferable heterogeneous feature subspace learning algorithm (THFSL). Both domain independent and domain related features are considered in the work where the transformation matrix is used to transfer the features from the source domain and target domain to a common feature subspace. Low ranking constrains imposed on the domain independent features help in the differentiation of stego image from the coverIn the paper, Solak et al.^20^ presents a performance evaluation of LSB Substitution and PVD. The parameters used in the comparison are—payload values, Peak Signal to Noise Ratio (PSNR) and Structural Similarity Index (SSIM). Results demonstrate that in LSB Substitution, the values for SSIM and PSNR are higher than the PVD algorithm and the PVD algorithm has better embedding efficiency with lesser visual perceptibility. Shankar et al.^21^ performs analysis of accuracy between SVM and SVM-PSO. The performance evaluation considers a minimum text embedding of 10% in JPEG images. Four various steganonographic algorithms- LSB Replacement, LSB Matching, PVD and F5 are used for the comparative study. The types of samplings-linear shuffled, stratified and automatic and kernels-linear, epanechnikov, multi-quadratic, radial, polynomial and ANOVA are used in the paper. Zou et al.^22^ proposed a steganalysis algorithm based on Markov model threshold prediction error image. The classifier used in the analysis is SVM. Detection rate is calculated for Piva et al.’s blind Spread Spectrum method, Cox et al.’s non-blind Spread Spectrum method, a Quantization Index Modulation method and LSB methods of various embedding rates.

Chaeikar et al.^23^ proposed a blind statistical technique for the detection of LSB image steganography. Pixel Similarity Weight was introduced in the work along with the filtering out of image pixels as per the statistically detected suspiciousness. An LSB Matching steganalysis on grayscale images is presented by Ker^24^. In the paper, Histogram Characteristic Function applied by calibrating the output and by calculating the adjacency histogram to overcome the previously drawbacks of HCF. In the paper, Che et al.^25^ proposed a novel steganalysis technique for the identification of binary image steganography. The techniques are based on Local Texture Pattern. Manhattan distance is used to amount the pixels correlation. The classifier used to differentiate stego and cover images is the ensemble classifier.

Features provide an important factor in determining the presence of a covert message^26^. Thus the selection of features had also become an element of paramount importance^27^. Pevny had brought forth the CC-PEV features to remove the problems faced by using either interblock features or intrablock features^28^. A classification with feature reduction using Principal Component Analysis had been done by Deepa et al.^29^ with a k-fold cross validation to evaluate the prediction. The validation had given impressive result when the fold used was 10^30^. Deepa had also done the study on moderate embedding of messages^31^. Zhang et al. had proposed a stratified validation model of face recognition using Support Vector Machine^32^. This research makes use of a large amount of data. Hence PCA is used for dimensionality reduction^33^. He et al. had used the PCA for accuracy enhancement^34^. However, Ma et al. had discussed the capacity of feature reduction using PCA and that the performance are better in linear systems^35^.

Many optimization algorithms have problems like misalignment and noise. This led to the use of metaheuristic method. The whale optimization algorithm (WOA) is one of them which had a balance between exploration and exploitation phases^36^. The disadvantage of this algorithm is that the complexity can be high for higher dimension problems^37^. Another evolutionary algorithm is Biogeography-based optimization (BBO). This disadvantage of this algorithm is its inability to exploit solutions and the inability to select the best member from each generation^38^. However, the advantage of Particle Swarm Optimization that is used in this paper are, easy in concept and coding implementation, limited parameters, impact of parameters to the solutions is less sensitive compared to other heuristic algorithms. PSO is less dependent of a set of points compared to other evolutionary methods. PSO techniques can also generate high-quality solutions with shorter calculation time and stable convergence characteristics^39–41^.

Support Vector Machine is a classifier used mainly for binary classification. It mainly works on the principle of kernels and optimal hyperplane^42^. The advantage of SVM is that it is faster for large sized problems and require less heuristics^43^. Hence SVM had been used as a mode of classification in this research.

## Problem statement

Recent research has paved ways to use machine learning as an effective medium of classification in the domain of steganalysis. Previous literature review with limited studies in image steganalysis have opened doors for the utilization of machine learning. Previous literature also shows that the statistical image steganalysis using machine learning had yielded better results. This work focus on performing blind steganalysis of random text embedding on images. In the proposed analysis, the JPEG images are transformed using Discrete Cosine Transform (DCT). Calibration is performed to get an estimation of the cover images. The following parameters are used for comparison:

1. *Domains* Spatial and Transform
2. *Steganographic algorithms* LSB Replacement, LSB Matching, PVD and F5
3. *Classifiers* SVM and SVM-PSO
4. *Kernels* Dot, Multiquadric, Epanechnikov, Radial and ANOVA
5. *Samplings* Linear, Shuffling, Stratified and Automatic

## Dataset

This work uses two datasets namely INRIA Holiday data set for training images and UCID dataset for testing images. These datasets together contain 2300 images and are in JPEG lossless format. JPEG lossless format was chosen due to its significance and the nature of work so that data or information is not misplaced during transmission. The size of the images used is 256 × 256. The dataset details are given in Table 1.

**Table 1:** Description of datasets used in the study.

Training images	Testing images	Image format	Image size
INRIA Holidays	UCID	Lossless JPEG	256 × 256
1500 images	800 images		

## Methodology

The lossless JPEG image is initially undergone pre-processing to ensure the image is free from noise and distortions. The preprocessing techniques used are the normalization and histogram equalization techniques. Further to the first step, the image is segmented using the block-based segmentation method where the image is split into blocks of size 8 × 8. The covert message is then embedded into the image using steganographic techniques of spatial or transform domain. Statistical features are then extracted from the stego image containing the text to be transmitted. The statistical features include—the first order features, the second order features and the Markovian features. Support Vector Classifier is used to differentiate stego and cover image. Particle Swarm Optimization is the optimization technique used in the paper.

In this paper, we consider and evaluate the performance of the steganographic algorithms from the spatial and transform domain. LSB Matching, LSB Replacement and Pixel Value Differencing methods are used to embed the text into the image. In case of frequency domain, the image is transformed using DCT before the F5 method is applied for text embedding. Random embedding percentages are used for embedding the data into the preprocessed image.

**Figure 2:**
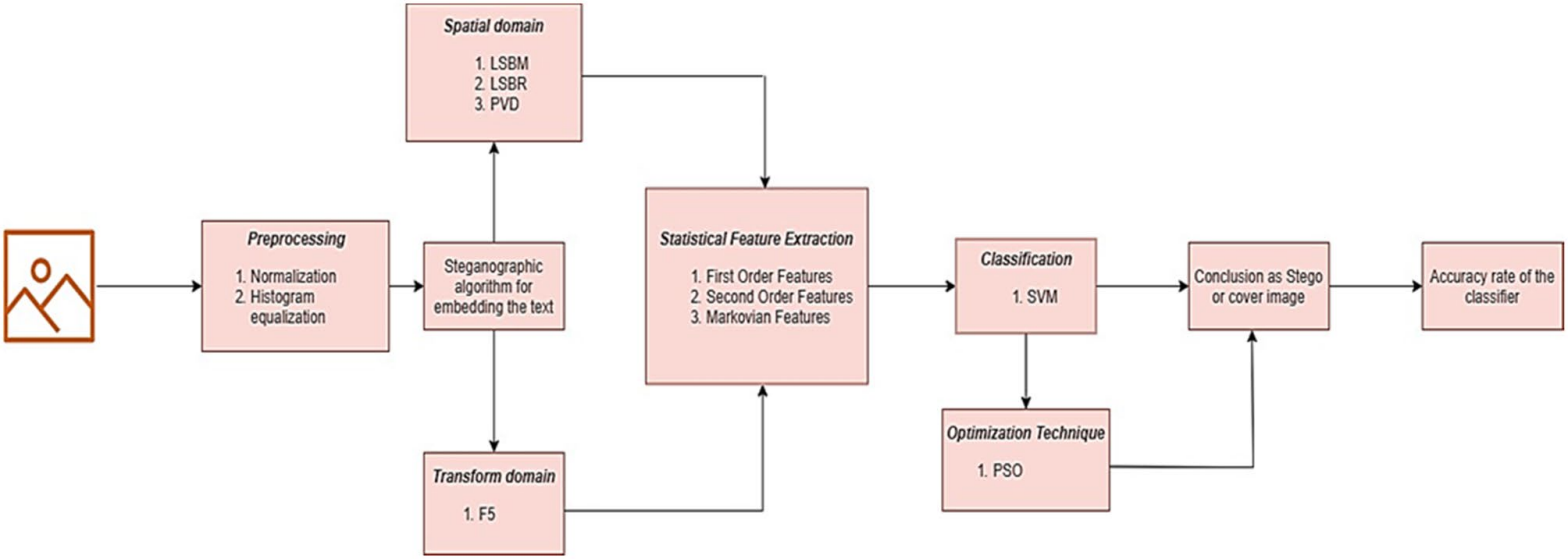
Flowchart explaining the methodology used in the work.

The methodology is clearly explained in the form of a flowchart in the Fig. 2.

### Implementation flow

Step 1: The image data is given as input.

Step 2: There is a choice of image undergoing calibration.

Step 3: To achieve a higher level of recognition, an efficient pre-processing method is used for the dataset. Two different image pre-processing techniques are used, they are normalization and histogram equalization. Normalization is used to resize the image into the correct size. The histogram is used to specify the intensity level.

Step 4: The steganographic algorithm is used to hide information in the image. The algorithms are used both in spatial and transform domains. The technique used for the spatial domain is LSB matching, LSB replacement, and PVD. The transform domain under research is the DCT and the technique used in the transform domain is F5.

Step 5: Once the message or data is hidden in the images, the feature selection followed by feature extraction is done. The techniques used for feature selection and feature extraction are first-order, second-order, extended DCT, and Markovian features.

Step 6: There is a choice of the use of cross-validation. With cross-validation, tenfold cross-validations have been done.

Step 7: Then sampling is done for the stego image using the technique like stratified sampling, linear sampling, shuffle sampling, and automatic sampling.

Step 8: SVM-PSO and SVM algorithms are trained using the algorithm and then tested. Once the algorithm is trained, the images are classified into a stego image and an ordinary image.

Step 9: The kernel takes the data as input and is converted into the required form.

### Preprocessing

Image processing is a technique done before image processing and model training. The objective of the preprocessing step is to curb the irrelevant distortions and to enhance the features essential for efficient image analysis. The techniques used in the paper for image preprocessing are—normalization and histogram equalization^44^.

#### Normalization

**Figure 3:**
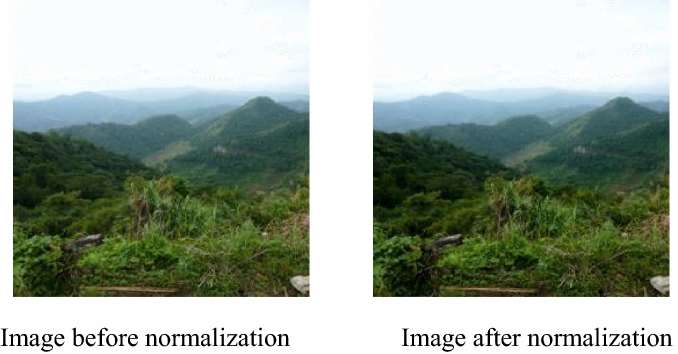
Images before and after normalization.

Normalization or histogram stretching/contrast stretching alters the range of the pixel intensity values for an improved assessment of the image. The images before and after normalization is shown in Fig. 3.

Let I be the image before preprocessing, let ${I}_{n}$ be the normalized image,${P}_{Minimum}$ and ${P}_{Maximum}$ be the minimum and maximum pixel intensity value of I which is the original image. ${N}_{Minimum}$ and ${N}_{Maximum}$ be the minimum and maximum pixel intensity value of ${I}_{n}$ which is the normalized image. Therefore, at location i, j , the normalized pixel intensity value is calculated using the Eq. (1)^44^.

1 $${I}_{n}\left(i,j\right)=\left(I\left(i,j\right)-{P}_{Minimum}\right)\frac{.{N}_{Maximum} - {N}_{Minimum}}{{P}_{Maximum}- {P}_{Minimum}}+{N}_{Minimum}$$

#### Histogram equalization

**Figure 4:**
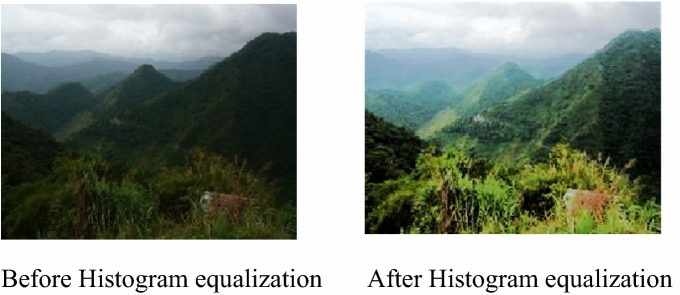
Images before and after Histogram equalization.

Histogram equalization is a technique that modifies the intensity histogram of an image to improve the contrast and dynamic range of the image^45^. The image before and after histogram equalization is as in Fig. 4.

Let g be the various gray levels in the image. The number of pixels under the various gray levels of the image is computer. This value helps to compute the cumulative histogram. To get the equalized pixel density value, the highest gray level value is multiplied to the cumulative histogram values and is rounded off to the nearest integer.

Let *P(X)* be the probability density where *X* is the gray level of the image and ${G}_{n}$ to be the number of pixels with gray level value. Let *n* denote the total number of pixels in the image. Then, the probability density of the image at gray level X is given by the Eq. (2).

2 $$\mathrm{P}(\mathrm{X})={G}_{n}$$

Let C(x) be the cumulative histogram at X and is denoted by the Eq. (3):

3 $$\mathrm{C}(\mathrm{X})=\sum_{P=0}^{X}P(X)$$

Let H(X) be the normalized histogram value at X and is denoted by the Eq. (4):

4 $$\mathrm{H}(\mathrm{X})=\frac{C(X)}{n(L-1)}$$

### Block-based image steganalysis

Several studies implement steganalysis on the whole image frame which involves difficulty in extraction of efficient features. Cho et al. proposed a method to evade this problem where the image is split into blocks consisting of N x N dimension. Each block is considered as an individual unit where feature extraction is performed. These features can used for classification to differentiate between the stego and cover image. The block based steganalysis is proved favorable as they show superior performance results without increase in the feature numbers. Block-based steganalysis also shows increased robustness and generalization capability of the classifier due to the generation of multiple samples during the splitting of the image into blocks^46^.

In this paper, the JPEG image is decomposed or split into blocks of 8 × 8 dimension.

### JPEG basics

**Figure 5:**
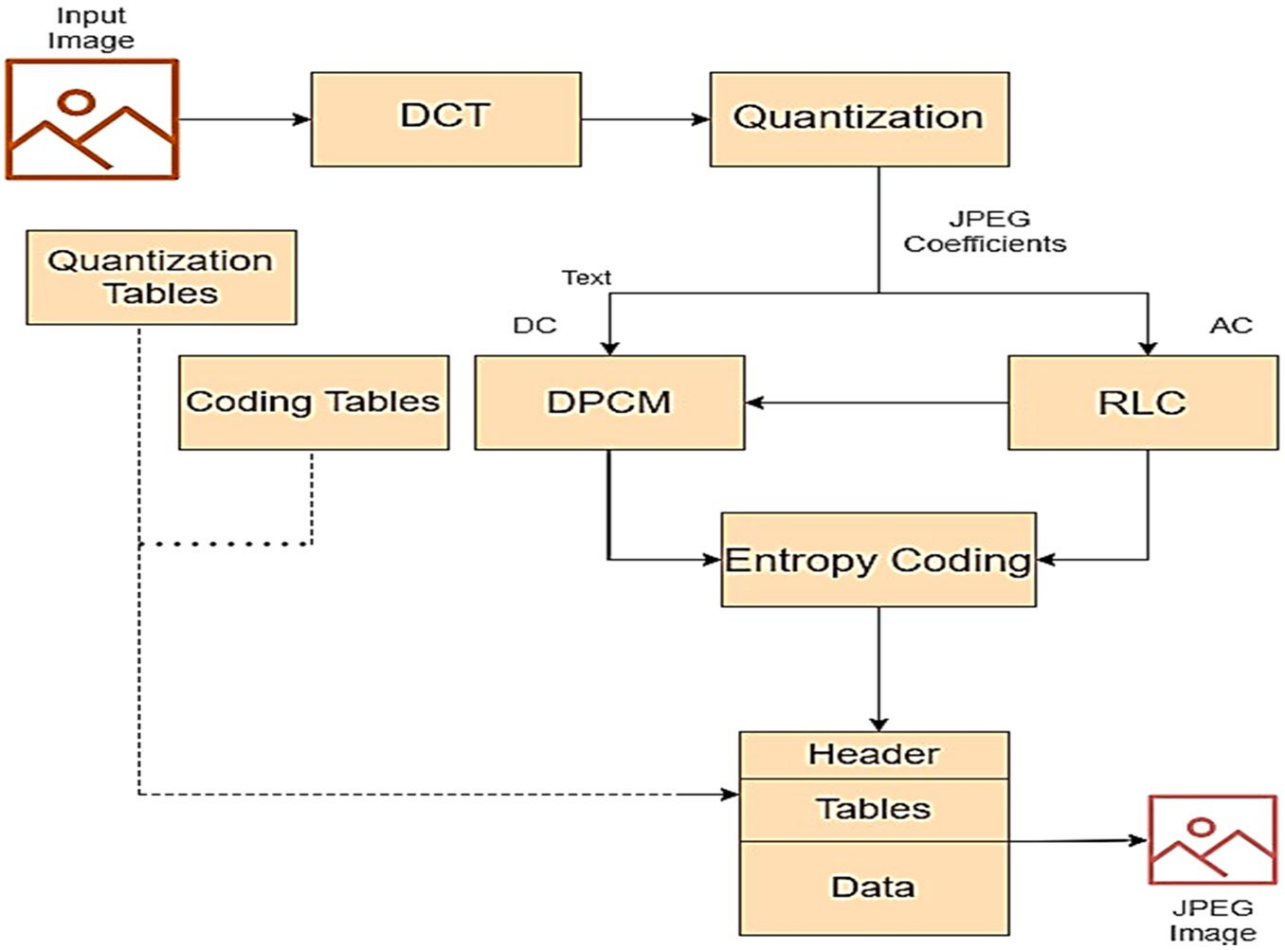
Block diagram of JPEG compression with lossy and lossless stages during the encoding process.

The wide used of JPEG or Joint Photographic Expert Group has made it a suitable medium for steganography. Several embedding algorithms have utilized JPEG and its properties making it fitting for steganalysis research^47,48^. JPEG algorithm is a competent compression algorithm^49^. The algorithm can also be used for non-lossy compression conditional to the compression ratio. The block diagram of JPEG compression is as in Fig. 5.

The steps of JPEG compression algorithm are as mentioned^50^:

1. Segmentation of the image into blocks: The image is initially decomposed into blocks of size 8 × 8 and of 64 pixels each. The further procedure is carried out on the individual blocks for improved efficiency.
2. Color space conversion: The RGB color space is then converted to the YCbCr color space. YCbCr uses two chroma components Cr and Cb for red difference and blue difference respectively and a luminance component Y.
3. Subsampling: Due to the special property of JPEG which used the previously mentioned YCbCr color space, subsampling is performed only on the chromas, and the luminance Y is left intact. This is because the luminance is more susceptible to detection by human eye than the chromas.
4. Transformation: The Y, Cb and Cr of each blocks undergo a transformation of DCT. DCT coefficients are calculated using the Eq. (5) where x, y are the coefficients post transformation and a, b is the pixel values prior transformation.
  5 $$I\left(x,y\right)=\frac{1}{4}C\left(x\right)C\left(y\right)\left[\sum_{a=0}^{7}\sum_{b=0}^{7}i\left(a,b\right)cos\frac{\left(2x+1\right)u\pi }{16}cos\frac{\left(2x+1\right)u\pi }{16}\right]$$
  where C(x) = C(y)=$\frac{1}{\sqrt{2}}$ if x = y = 0 and C(x) = C(y) = 1 otherwise.
5. Quantization: Quantization helps the high frequencies to get close to 0 and to preserve the low frequencies in the coefficients.
6. Zig-Zag ordering and final lossless compression is done to compress the remaining data. This is advantageous during message embedding in least significant bits due to its visual faintness.

Embedding in JPEG images is done by changing DCT coefficient values to reflect the message. After the embedding process, the altered coefficients are compressed using entropy encoding to create stego image.

### Steganographic algorithms

The steganographic algorithms used in the work is classified into two main domains: spatial domain and transform domain.

#### Spatial domain

In spatial domain, the secret message is embedded directly on the image pixel value of the cover image. x_s_ in the spatial domain is denoted as the analog samples in discrete form. Images in the spatial domain is represented as a matrix intensity measurement arranged at an equal distance. Spatial domain vulnerable to formatting and is robust only to lossless compressed files. The paper uses PVD and LSB techniques, which can be further sub-classified as LSB Matching and LSB Replacement.

#### Least significant bit replacement (LSB replacement)

LSB Replacement is one of the earliest data hiding techniques in image steganography^51^. The LSB technique is based on the concept that the least significant bit in a digitalized signal is noisy making the data hiding less prone to detection. The LSB plane is random and is difficult to differentiate from LSB plane of a stego image. Thus, making the algorithm a powerful implementation in both spatial domain^52^ and DCT domain^53^. The message to be concealed is embedded by replacing the LSB of the image byte sequentially or with the help of a pseudo random key^1,54^. LSB Replacement can be represented using the Eq. 6

6 $${{x}_{s}}^{(1)}\leftarrow 2\left[{{x}_{s}}^{(1)}/2\right]+{m}_{j}$$

While using implementations using the transform domain as in this research, LSB replacement skips the coefficient with values ${x}^{(0)}\epsilon \left\{\mathrm{0,1}\right\}$. This is to stop the percerptible artefacts from changing many of the 0 s to 1 s. Many of the steganographic algorithms are detected using the intrusion detection systems. Hence, miscreants may resort to typing the message manually into LSB to avert being caught.

#### Least significant bit matching (LSB matching)

LSB matching^55^ is a simple form of steganography like LSB replacement but difficult to detect in spatial domain images^56,57^. This method of embedding compares the message to be embedded in a least significant bit and modifies it. In this type of embedding, LSB is modified^58^ rather than flipped. The adjustment is such that the bits are arbitrarily raised or decreased. Hence, LSB matching steganalysis is more difficult than LSB replacement^59^. The modification works this way:

- The message to be hidden is converted to bits.
- Every pixel of the cover image is taken in a shared key generated pseudorandom order
- If the next cover pixel’s LSB matches the next message bit, then do nothing.
- Else add or subtract one from cover pixel randomly.
- If the message length is less than the cover pixel length of the cover image, the pseudorandom permutation allows the changes to be uniformly spread across the image.

The allowable range of pixel value decides the increase or decrease in the values.

#### Pixel value differencing (PVD)

Wu and Tsai proposed the Pixel Value Differencing method^60^. This is used in the transform domain, mainly for block-based steganography. This embedding algorithm is also used as a part of research here. This is selected because the algorithm uses PRNG to achieve secrecy just as NsF5. It works as follows:

- A difference value from two pixels in a block is calculated.
- All possible values are classified into different ranges.
- The difference value will be replaced by the message bit. However, it will be ensured that the bit will not go beyond the range.

A small difference of value would indicate that the block is in the smooth area and a larger difference of value indicates that the block is in the edge area. This method helps to retrieve an imperceptible result than LSB replacement.

##### Transform domain–discrete cosine transform

In transform domain image processing, the DCT places a prominent position. It adds to the compression method of the JPEG^61^. The DCT converts intensities of spatial pixels to coefficients of alternate current (AC) and direct current (DC). The DCT image size of N × N image size is represented as the Eqs. (7 and 8).

7 $$\mathrm{C}\left(u,v\right)=\mathrm{\alpha }(v)\sum_{x-0}^{N-1}\sum_{y-0}^{N-1}\mathrm{f}(x,y)\mathrm{cos}\left[\frac{(2x+1)u\uppi }{2N}\right]\times \mathrm{cos}\left[\frac{(2x+1)u\uppi }{2N}\right]$$

8 $$\mathrm{\alpha }\left(u\right)=\left\{\begin{array}{c} \frac{1}{N} , for u=0; \\ \frac{\sqrt{2}}{N},for u=\mathrm{1,2},\dots ,N-1\end{array}\right.$$

DCT converts the image or signal into the frequency domain from the spatial domain. The image is split into 8-pixel blocks and the pixel blocks are transformed into 64 DCT blocks. The DCT is implemented to each block from the left to right, up to down. Each block is compressed by the quantization table and the message is inserted in coefficients of DCT. The image is reconstructed by decompression when needed, a process that uses the inverse DCT that is, IDCT^62^. Once DCT is performed, the quantization is applied to eliminate the components of high frequency.

9 $${\mathrm{DCT}}_{\mathrm{quantized}}=\mathrm{Round }\left(\frac{{\mathrm{DCT}}_{\mathrm{Coefficients}}}{{\mathrm{Q}.\mathrm{Scale}}_{\mathrm{Factor}}}\right)$$

The quantized DCT in Eq. (9) is obtained by dividing the coefficient DCT value by quantized scale factor and rounding it off, where ${\mathrm{DCT}}_{\mathrm{quantized}}$ is used in the image processing. The DCT quantization is obtained by compressing a values range to a single quantum value.

${\mathrm{Q}\cdot \mathrm{Scale}}_{\mathrm{Factor}}$—Scaling is unpredictable in many applications like JPEG, so, a scale factor is combined with the corresponding computational quantization step in JPEG and a scaling enables the DCT to be calculated with fewer multiplications.

The steganographic algorithm that makes use of the DCT and used in this research is F5.

#### F5 Embedding

F5 is a transform domain embedding proposed by Andreas Westfeld^63^. The F5 uses matrix-embedding coding which decreases the number of embedding changes, especially for smaller payloads. The working of F5 is as follows^64^.

- Get the RGB representation of the image
- Calculate the quantization table corresponding to quality factor Q and compress the image while storing the quantized DCT coefficients.
- Compute the estimated capacity with no matrix embedding C = h_DCT_ − h_DCT_ /64 − h(0)  − h(1) + 0.49 h(1), where h_DCT_ is the number of all DCT coefficients, h(0) is the number of AC DCT coefficients equal to zero, h(1) is the number of AC DCT coefficients with absolute value 1, h_DCT_/64 is the number of DC coefficients, and − h(1) + 0.49 h(1) = − 0.51 h(1) is the estimated loss due to shrinkage. The parameter C and the message length together determine the best matrix embedding.
- The user-specified password is used to generate a seed for a PRNG that determines the random walk for embedding the message bits.
- The message is divided into segments of k bits that are embedded into a group of 2^k^–1 coefficient along the random walk.
- If the message size fits the estimated capacity, the embedding proceeds, otherwise an error message showing the maximal possible length is displayed.

Since the F5 is based on matrix encoding, LSB of DCT coefficients is not flipped directly but decreased. Hence, the overall histogram is preserved after embedding. This effect is called shrinkage since DCT coefficients are reduced to zero.

### Features for extraction

The research involves statistical steganalysis, where features are selected, extracted, and analyzed. There are many features in an image. The features that are relevant and show a significant change in behavior when an embedding is done are selected. The research makes use of four relevant feature sets-first-order, second-order, extended DCT, and Markov features. When the DCT features capture the interblock dependency, the Markov features capture the intrablock dependencies. This merge helps to eliminate the drawbacks of inter- and intrablock dependencies taken individually. Different features are extracted from the frequency. A few are explained below:

#### Global histogram

**Figure 6:**
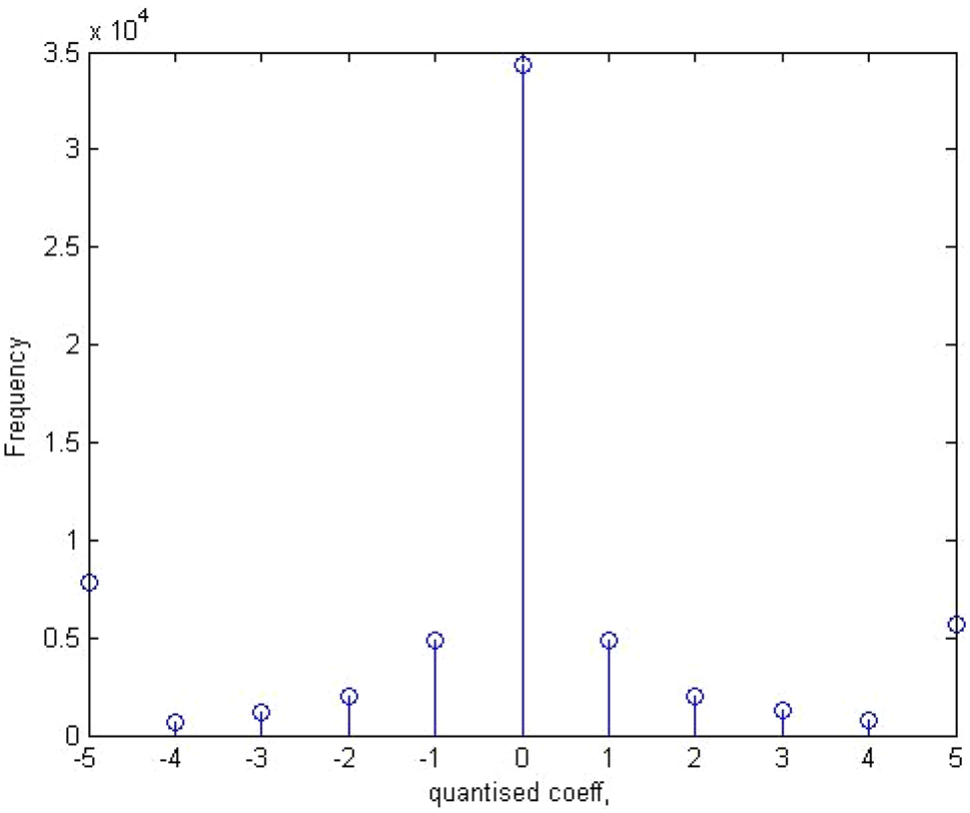
Global histogram of sample stego image.

**Figure 7:**
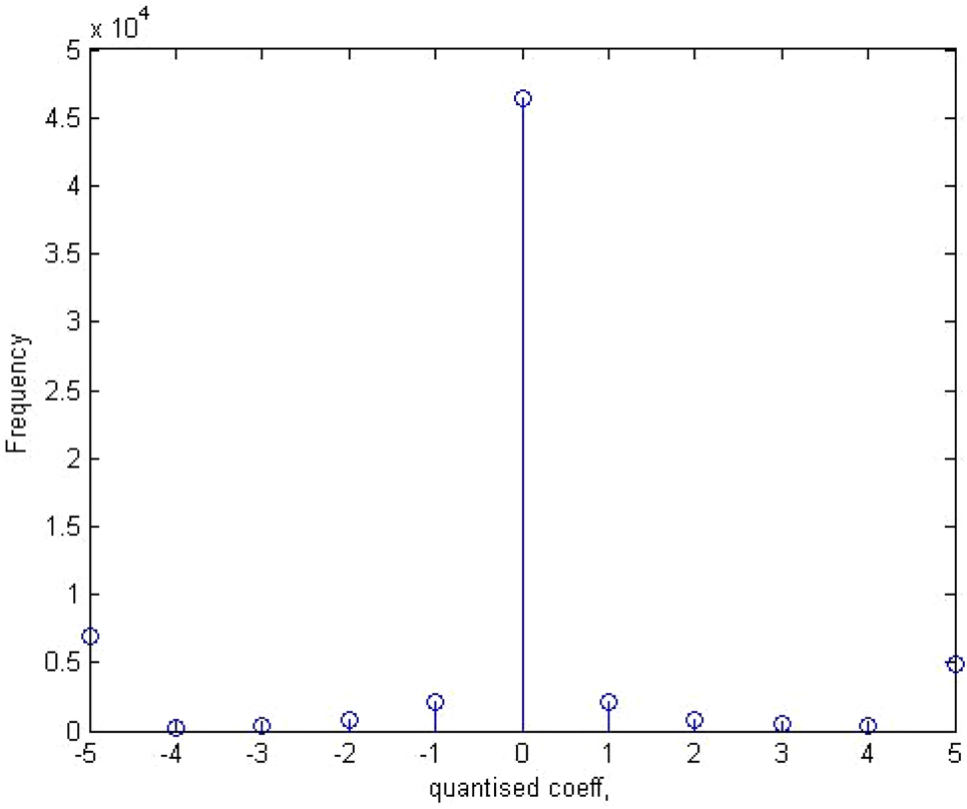
Global histogram of sample calibrated stego image.

This histogram gives an idea of the total number of times a particular coefficient would appear in the entire image in all the blocks in the transform. Assume that a stego image is represented by DCT coefficient array *d*_*k*_(*i*,*j*), where *i* and *j* are coefficients and *k* is the block. The global histogram of 64 k blocks can be represented by Gr where *r* = A,,B, A = min _*k*,*i*,*j*_ (*d*_*k*_(*i*,*j*)), B = max _*k*,*i*,*j*_ (*d*_*k*_(*i*,*j*)). Figures 6 and 7 below give an overview of the global histogram of a sample stego image and its equivalent in a calibrated stego image.

The global histogram used in the research is normalized to 11 features. The features are normalized from -5 to + 5.

#### Dual histogram

The dual histogram shows the total number of times a particular coefficient appears in the entire image at specific locations. It is represented by the Eq. 10.

10 $${g}_{ij}^{d}={\sum }_{k=1}^{B}x\left(d,{d}_{k\left(i,j\right)}\right)$$

where *B* is the number of blocks. They are represented by 11 functionals.

**Figure 8:**
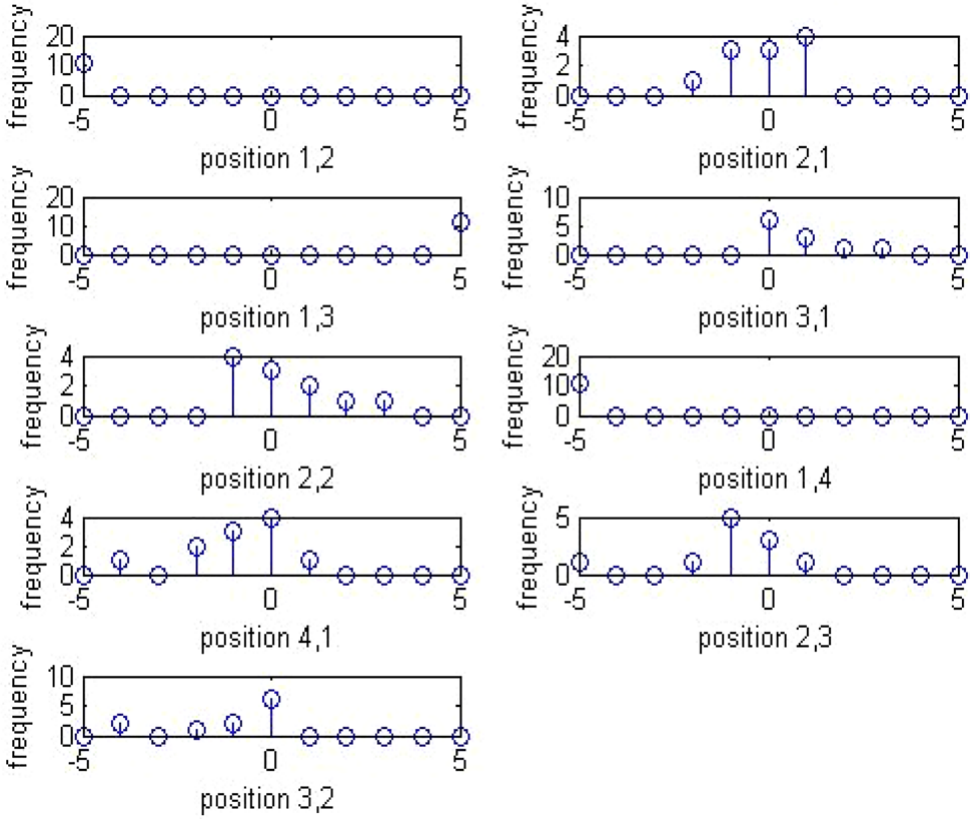
Dual histogram of sample stego image.

**Figure 9:**
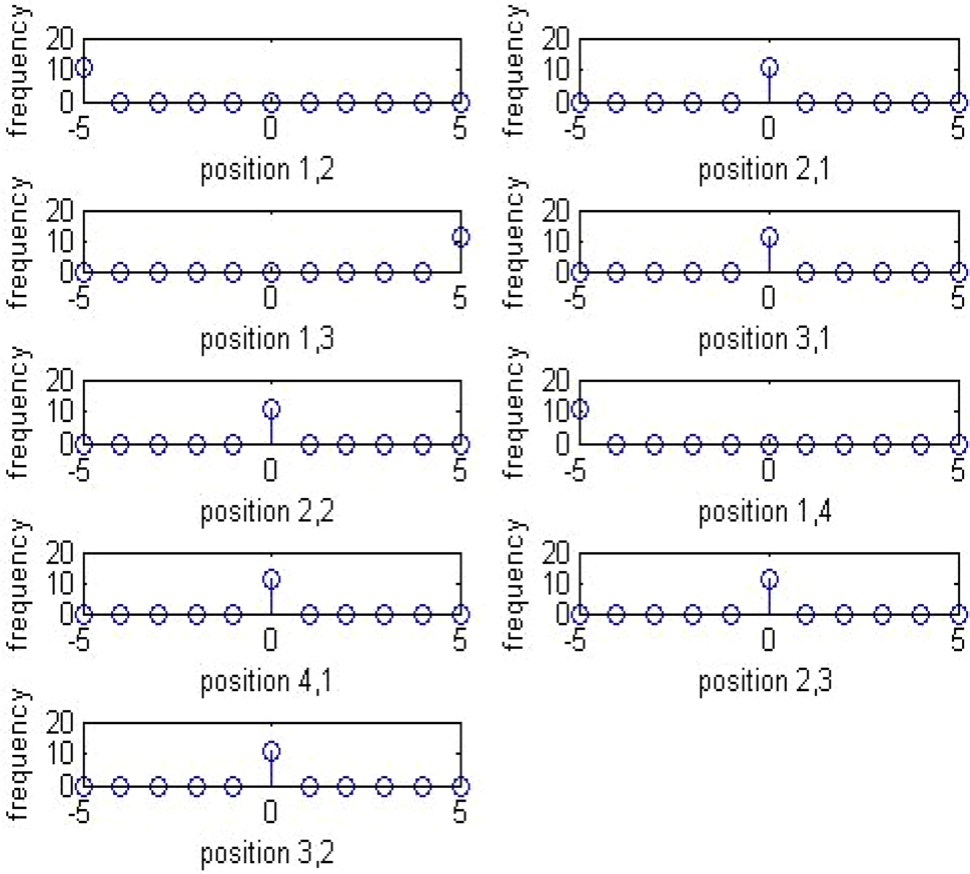
Dual histogram of sample calibrated stego image.

The locations are the lowest AC frequency coefficients in a transformed image. Figures 8 and 9 below give an overview of the dual histogram of a sample stego image and its equivalent in a calibrated stego image.

For dual histogram of 99 functionalities, the 9 upper triangular values are considered.

F5 is a transform domain embedding proposed This feature measures the variation of DCT coefficients among different blocks, both row wise and column wise. The variance is calculated by the following Eq. (11). Hence this feature captures the interblock dependencies.

11 $$V=\frac{\sum_{{\varvec{i}},{\varvec{j}}=1}^{8}\boldsymbol{ }\sum_{{\varvec{k}}=1}^{\left|{{\varvec{I}}}_{{\varvec{r}}}\right|-1}\boldsymbol{ }\left|{{\varvec{d}}}_{{\varvec{I}}{\varvec{r}}\left({\varvec{k}}\right)\left({\varvec{i}},{\varvec{j}}\right)\boldsymbol{ }}-{{\varvec{d}}}_{{\varvec{I}}{\varvec{r}}({\varvec{k}}+1)({\varvec{i}},{\varvec{j}})}\right|+\sum_{{\varvec{i}},{\varvec{j}}=1}^{8}\boldsymbol{ }\sum_{{\varvec{k}}=1}^{\left|{{\varvec{I}}}_{{\varvec{c}}}\right|-1}\boldsymbol{ }\left|{{\varvec{d}}}_{{\varvec{I}}{\varvec{c}}\left({\varvec{k}}\right)\left({\varvec{i}},{\varvec{j}}\right)}-{{\varvec{d}}}_{{\varvec{I}}{\varvec{c}}({\varvec{k}}+1)({\varvec{i}},{\varvec{j}})}\right|}{\left|{{\varvec{I}}}_{{\varvec{r}}}\right|+\left|{{\varvec{I}}}_{{\varvec{c}}}\right|}$$

where *I*_*r*_ and *I*_*c*_ are block index vectors, when scanned by rows and columns. Variance captures the interblock dependency of various DCT coefficients.

#### Blockiness

Blockiness is an interblock dependency feature. This is calculated in decompressed DCT images. The logic of blockiness is to find the sum of boundaries calculated both row wise and column wise, and a difference in each value for cover and stego images is calculated. The blockiness is represented by the Eq. (12).

12 $$B_{\alpha } = ~\frac{{\sum\nolimits_{{i = 1}}^{{\left| {(M - 1)/8} \right|}} ~ \sum\nolimits_{{j = 1}}^{N} {\left| {x_{{8i,j}} ~ - ~~x_{{8i + 1,j}} ~~~} \right|^{n} } ~ + ~\sum\nolimits_{{i = 1}}^{{\left| {\left| {(N - 1)/8} \right|} \right|}} ~ \sum\nolimits_{{j = 1}}^{M} {\left| {x_{{8i,j}} ~ - ~~x_{{8i + 1,j}} ~~~} \right|^{n} } }}{{N\left\lfloor {(M - 1)/8} \right\rfloor + M\left\lfloor {(N - 1)/8} \right\rfloor }}$$

where *M* and *N* are dimensions of the image.

#### Co-occurrence

The probability distribution of pairs of neighboring DCT coefficients can be determined with the help of the co-occurrence matrix. It measures the extent of which two DCT coefficients occur together in an image. Co-occurrence is measured in terms of the Eq. (13).

13 $${C}_{st}=\frac{\sum_{k=1}^{\left|{I}_{r}\right|-1}\sum_{i,j=1}^{8}x(s,{d}_{i,\left(k\right)}\left(i,j\right))x(t,{d}_{i,\left(k+1\right)}(i,j)+\sum_{k=1}^{\left|{I}_{c}\right|-1}x(s,{d}_{i,\left(k\right)}\left(i,j\right))x(t,{d}_{i,\left(k+1\right)}(i,j)}{\left|{I}_{r}\right|+\left|{I}_{c}\right|}$$

**Figure 10:**
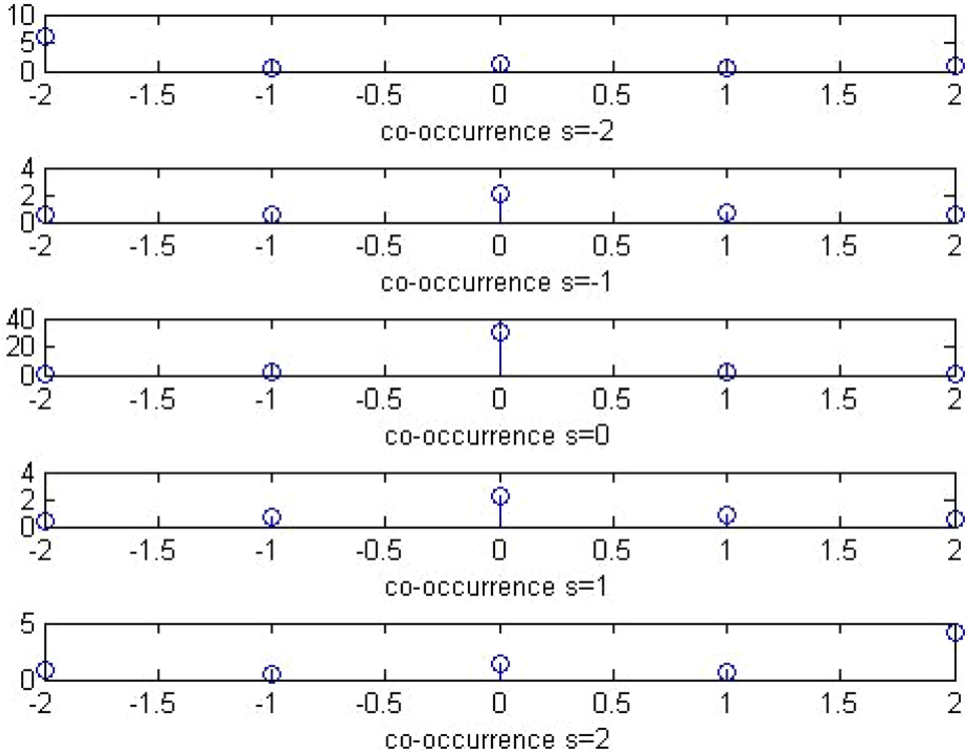
Plot of co-occurrence matrix of stego image.

**Figure 11:**
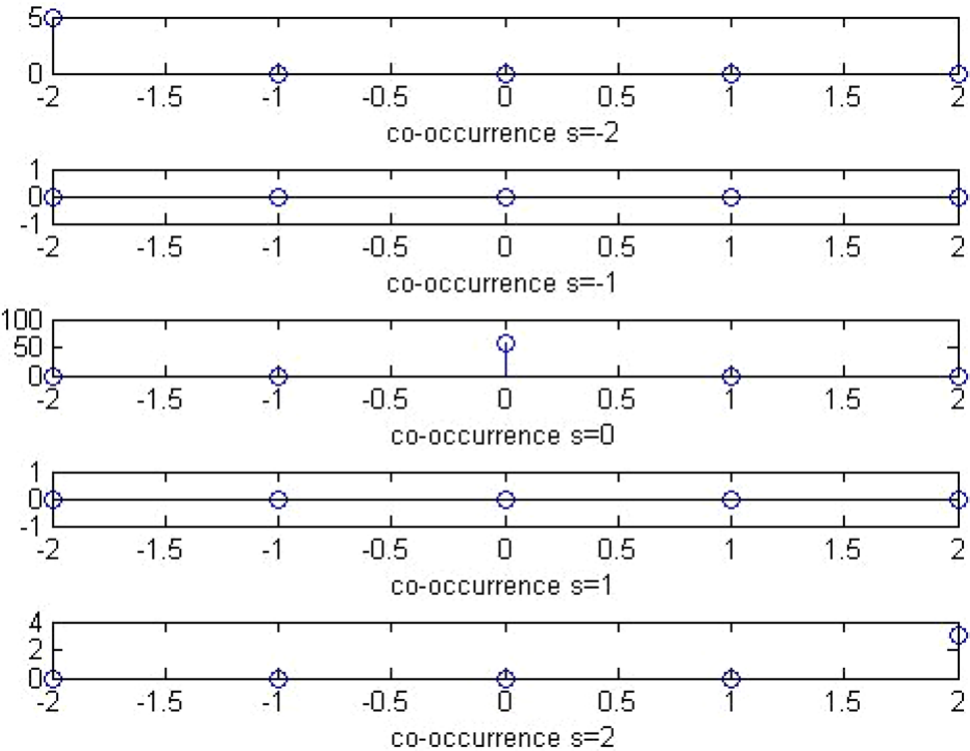
Plot of co-occurrence matrix of calibrated stego image.

A sample plot of the co-occurrence matrix is shown below Figs. 10 and 11:

Thus, the extended DCT features will be of 193 features. The extended feature set will be the global histogram of dimensionality 11,5 AC histogram with 11 dimensionality which would mount up to 55,11 dual histograms with 9 significant features taken, which would lead to 99, variation feature of 1, blockiness feature of 2 and co-occurrence feature of 25. These functionals are retrieved as follows. For global histogram, the difference of elements from [-5, 5] is taken which would sum up to 11 features. For dual histogram, the difference of the lowest 9 AC modes is used. They are (2,1), (3,1), (4,1), (1,2), (2,2), (3,2), (1,3), (2,3), and (1,4). For the functionals for co-occurrence matrix, the central elements in the range [− 2,2] × [− 2,2] is used which yield 25 features.

#### Markovian features

This involves modelling the JPEG coefficients as Markov process and the features are extracted from it. It has intrablock dependencies between the DCT coefficients. The Markov feature set is generated by computing four difference matrices from a quantized 2D array of a JPEG image taken along four directions-horizontal, vertical, diagonal major, and diagonal minor directions.

The Markov features can be calculated from the four-difference matrices as per the Eqs. (14,15,16 and 17).

14 $${F}_{h}\left(u,v\right)=F\left(u,v\right)-F\left(u+1,v\right)$$

15 $${F}_{v}\left(u,v\right)=F\left(u,v\right)-F\left(u,v+1\right)$$

16 $${F}_{d}\left(u,v\right)=F\left(u,v\right)-F\left(u+1,v+1\right)$$

17 $${F}_{m}\left(u,v\right)=F\left(u+1,v\right)-F\left(u,v+1\right)$$

where *F*(*u*,*v*) is a quantized DCT coefficient, *u ∈ *[1,*R*_*u*_* − *1], *v ∈ *[1,*R*_*v*_* − *1], *Ru* is the 2D array size of the JPEG in horizontal direction, *R*_*v*_ is the array size in vertical direction, *F*_*h*_*, F*_*v*_*, F*_*d*_*, F*_*m*_ are the difference arrays in horizontal, vertical, major, and minor diagonals, respectively.

From Eqs. (18,19,20 and 21), the actual transition probability matrices are created.

18 $${M}_{h}\left(j,k\right)= \frac{\sum_{x=1}^{N-2}\sum_{y=1}^{M}{\partial }_{{F}_{h}}\left(x,y\right),j{\partial }_{{F}_{h}}\left(x+1,y\right),v}{\sum_{x=1}^{N-1}\sum_{y=1}^{M}{\partial }_{{F}_{h}}\left(x,y\right),u}$$

19 $${M}_{v}\left(j,k\right)= \frac{\sum_{x=1}^{N}\sum_{y=1}^{M-2}{\partial }_{{F}_{v}}\left(x,y\right),j{\partial }_{{F}_{v}}\left(x,y+1\right),v}{\sum_{x=1}^{N}\sum_{y=1}^{M-1}{\partial }_{{F}_{v}}\left(x,y\right),u}$$

20 $${M}_{d}\left(j,k\right)= \frac{\sum_{x=1}^{N-2}\sum_{y=1}^{M-2}{\partial }_{{F}_{d}}\left(x,y\right),j{\partial }_{{F}_{d}}\left(x+1,y+1\right),v}{\sum_{x=1}^{N-1}\sum_{y=1}^{M-1}{\partial }_{{F}_{d}}\left(x,y\right),u}$$

21 $${M}_{m}\left(j,k\right)= \frac{\sum_{x=1}^{N-2}\sum_{y=1}^{M-2}{\partial }_{m}\left(x+1,y\right),j{\partial }_{m}\left(x,y+1\right),v}{\sum_{x=1}^{N-1}\sum_{y=1}^{M-1}{\partial }_{{F}_{m}}\left(x,y\right),u}$$

To reduce the dimensionality, an average of all four matrices are taken and the resulting value of features will be 81.

Table 2 gives a list of features extracted and its dimensionality.

**Table 2:** Table of extracted features.

	Feature extracted	Number of Features Extracted
First order features	Dual histogram	99
	Global histogram	11
	Individual histogram	55
Second order features	Co occurrence	25
	Blockiness	2
	Variance	1
Markovian features		81
Total number of features extracted		

### Calibration

**Figure 12:**
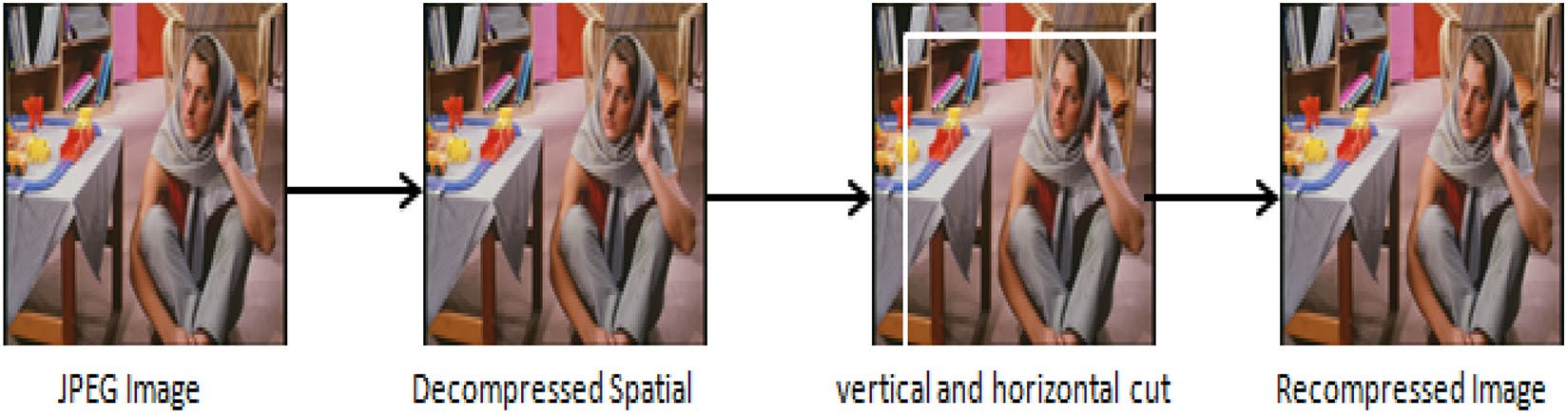
Diagram explaining the calibration process.

The characteristics of the cover image can be estimated by the process of calibration. Fridrich calibrated the image by cropping the horizontal and vertical pixels by 4^65^. During the calibration process, the stego JPEG image is decompressed into the transform domain. As the stego image, it is then cropped again with the same quality matrix. The process of calibration is as shown in the Fig. 12.

Once both images are available, the characteristics of the image can be compared with the L1 norm. However, considering the calibration, the features may show significant improved performance and hence taken into consideration in the research.

### Classification

Once the features are extracted from the transformed domain, it needs to be classified. The classification techniques can be broadly classified as supervised learning and unsupervised learning. In this research, supervised learning is considered. A steganalytic algorithm takes in an image and decides whether it is a clean image (cover image) or has a message embedded in them (stego image). The thesis looks at the steganalysis method as a binary classification problem. The classification is explained in^66^. For the proposed research, the two phases-training and testing phases, are being considered for the design of the classifier.

#### Training phase

During this phase, the classification algorithm is given a set of data that is labeled as either a stego or a cover. This applies to images that is an already known stego or cover image. For research, the labelled cover and stego images are required to be available. There are innumerable standardized datasets available, which can be used for research. The research takes care of the lower embedding rates as well. This represents the best-case scenario, which is difficult to achieve in real life.

#### Testing phase

Once the training phase is complete, the testing phase should be done on the classification system. For a clear division, the testing dataset should be different from the training dataset since the analysis in real life is always done with unseen data. This decision makes sure that the classifier will not be over learnt.

#### Support vector machine

**Figure 13:**
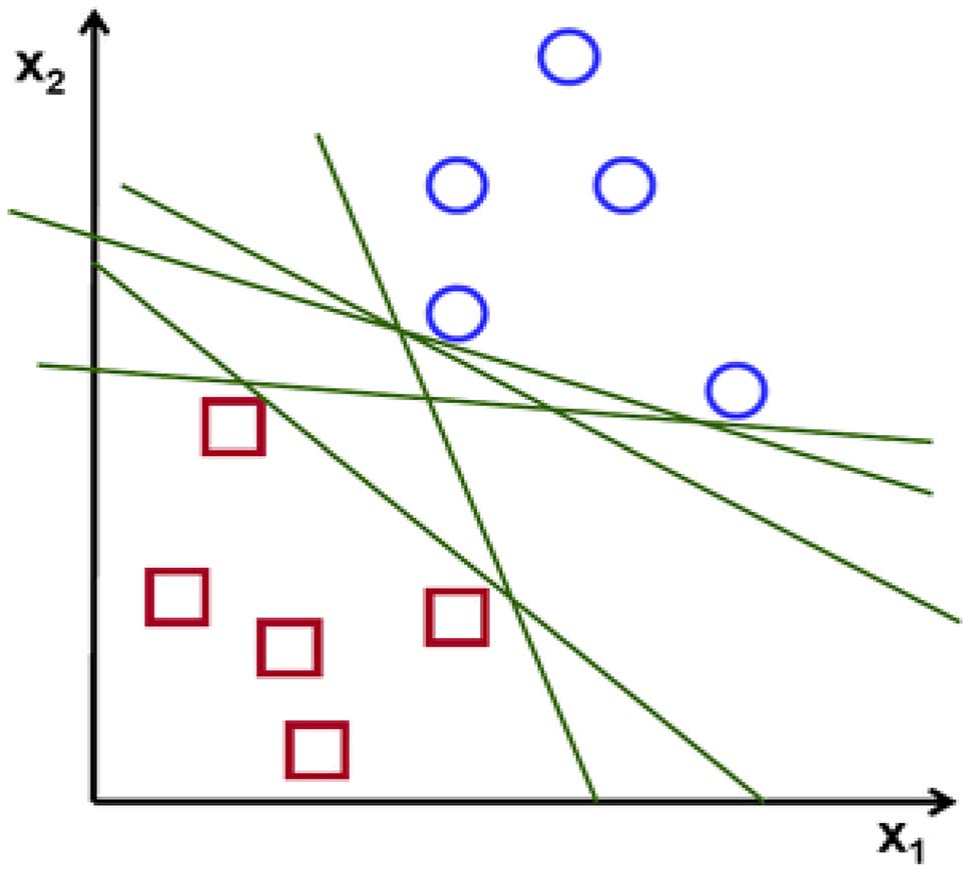
Depiction of two classes and various hyperplanes to provide optimal solution.

Support Vector Machines (SVM) by Vapnik^67^ is the common pattern classifier used for binary classification^68^. The classification is done in SVM by creating a hyperplane or a set of hyperplanes, if the values are in a high dimension as in Fig. 13.

The goal of SVM is to find an optimal hyperplane that gives the biggest least distance to training samples. Thus, the optimal separating hyperplane maximizes the boundary of the training set of data.

Consider *x*_*i*_ to be a point and *w* be the weight. Both values are vectors. To separate the data using Eq. (22),

22 $$w^{\prime}x_{i} + b \ge 1$$

Among all the hyperplanes, SVM selects the one in which the hyperplane is as large as possible. SVM belongs to a family of generalized linear classifiers.

It is improbable to get an exact separate line that divides the data into the space. However, a curved decision boundary may be available. Thus, *y*_*i*_ (*w*’*x*_*k*_ + *b*) ≥ 1 − *S*_*k*_. This permits a point to be a small distance S_*k*_ on the incorrect side of the hyperplane without violating the check rule. This can have enormous slack variables, which permit any line to isolate the data. Large slacks can be eliminated using lagrangian variables in Eq. (23).

23 $${\text{Min}}\,L \, = 1/2w^{\prime}w - \sum \, \lambda_{k} (y_{k} \left( {w^{\prime}x_{k} + b} \right) + s_{k} - 1) + \alpha \sum s_{k}$$

where limiting *α* allows more data to lie on the wrong side of the hyperplane and would be considered as outliers which give flatter decision boundary. A separating hyperplane is used to split the data if the data is linear in function.

**Figure 14:**
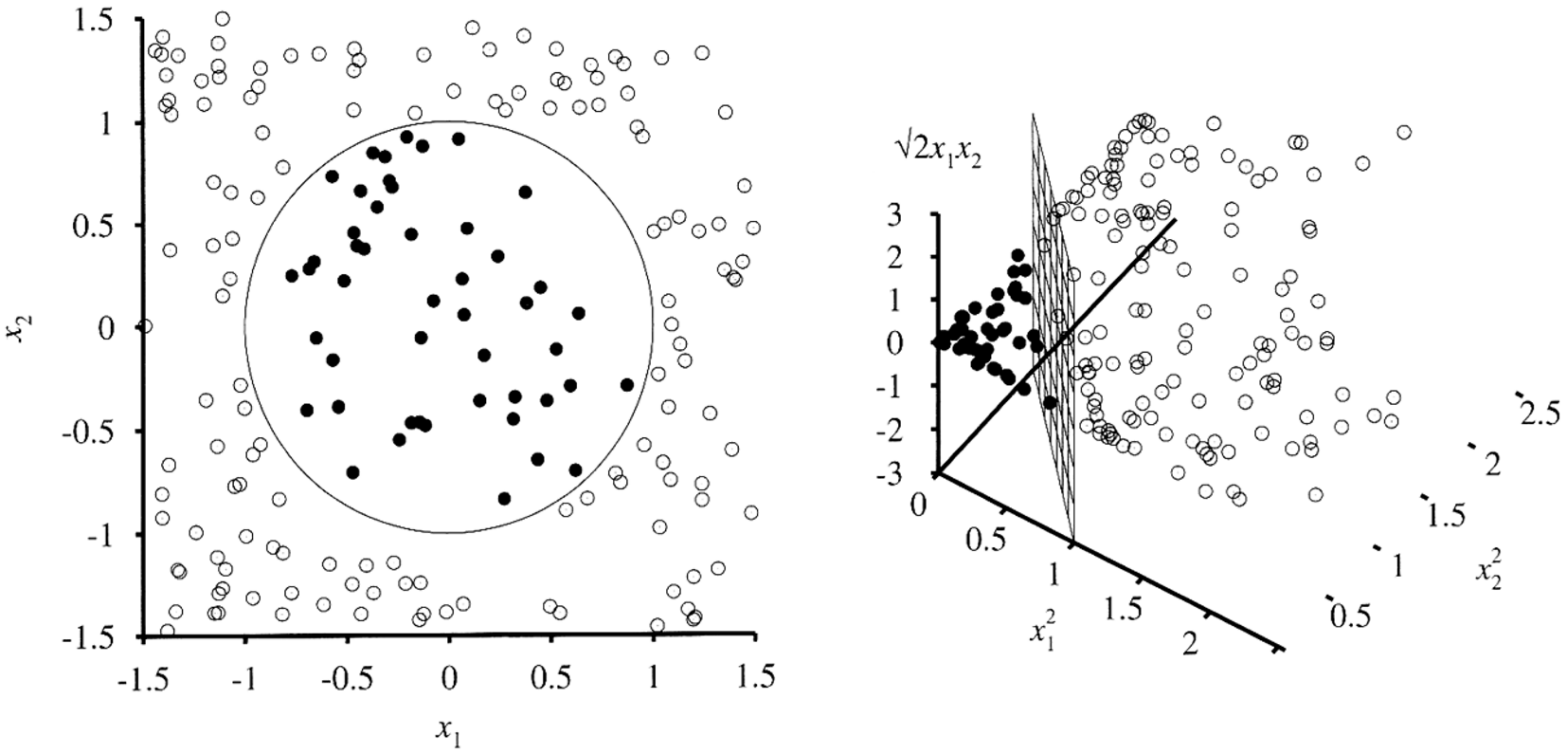
Feature space representation.

Transforming the data into feature space enables a measure of similarity to be claimed based on the dot product. The feature space is as depicted in Fig. 14. Recognition of patterns can be simple if the feature spaces are carefully chosen suitably.

When *w*, *b* is retrieved; the solution is obtained for a data that is divided by a hyperplane. This helps the SVM to create nonlinear boundaries. Algorithm involved in kernel trick are given below.

1. The algorithm is represented as the inner products of datasets. This is known as the dual problem.
2. Original data are passed through nonlinear maps to create new data. This is created from new dimensions by accumulating a pairwise product of some of the original data dimension to each data vector.
3. A dot product of the data can be represented after doing nonlinear mapping on them. SVMs had been considered in the research due to many factors.

They display excellent performance when faced with nonlinear problems^69,70^. SVM is a popular classifier for high-dimensional data because of its reduced sensitivity to dimensionality and robustness to noisy data^71^.

#### Support vector machine with particle swarm optimization

A comparative study may be done using different classifiers to arrive at a conclusion on the effectiveness of each. SVM with particle swarm optimization (PSO)^72^, is used for a comparative analysis. PSO was first proposed in 1995^73^ and has become increasingly popular due to its flexibility, low computational requirement, and easy implementation. The PSO is an evolutionary model of computing dependent on swarm intelligence^74,75^. In PSO, the group of birds is called particle, which will form a population in a D-dimensional feature space^76^.

The algorithm for parameter selection of PSO in SVM^76^ will be as follows:

1. Initialize the parameters. They can be maximum iteration number, population, etc. Determination of the initial velocity *v*_*i*_^0^ and location *x*_*i*_^0^ must be done. *X* = rand (− *x*_id max_, *x*_idmax_) will be determined next, where rand() is a random function in the range [0,1]. Set gen = 1.
2. A set of c and r is generated randomly. Each selected kernel function and parameters are considered as individual parameter of SVM. Thus, the evolution starts.
3. Calculate the new position v_i_0 and x_i_0 of the i-th particle. Input x_i_ into the SVM model for forecasting. The f_i_, k-fold cross-validation, is then calculated.
4. The offspring generation is done. The best global value is generated from the Eqs. 24 and 25 given below:
  24 $${v}_{id}^{t+1}=\omega \cdot {v}_{id}^{t}+{c}_{1}\cdot {r}_{1}\cdot \left({p}_{id}-{x}_{id}^{t}\right)+{c}_{2}\cdot {r}_{2}\cdot ({p}_{gd}-{x}_{id}^{t})$$
  25 $${x}_{id}^{t+1}={x}_{id}^{t}+{v}_{id}^{t+1}$$

where V_i_ = (vi_1_,vi_2_,vi_3_….vi_D_) is the velocity of the i-th particle, Pi = (pi_1_,pi_2_,pi_3_….pi_D_) is the optimal position of this particle. The optimum swarm position is Pg = (pg_1_, pg_2_, pg_3_….pg_D_). when the i-th particle is at the t-th iteration, xtid and ytid are the d-th location and velocity. c_1_, c_2_, r_1_, r_2_ are random numbers which has value that range from 0 to 1 and ω is the inertial weight of the PSO algorithm.

26 $${\text{Set}}\;{\text{gen}} = {\text{gen}} + 1$$

5. When gen is equal to the maximum number of iterations, the circulation will be stopped, thus obtaining the optimal parameters of SVM.
6. The PSO algorithm helps in optimization, thus improving the performance when teamed with SVM. PSO^77^ with SVM has been taken into consideration in the research.

### Cross-validation

The cross-validation techniques is a general scheme to estimate the prediction error of any supervised learning classifier^78^. Numerous preparation and testing are conducted. During the training process, the training set is divided into *k* equally populated folds. The training is done on *k*-1 folds and evaluated on the remaining fold. This is repeated *k* times, using every fold for error estimation.

**Figure 15:**
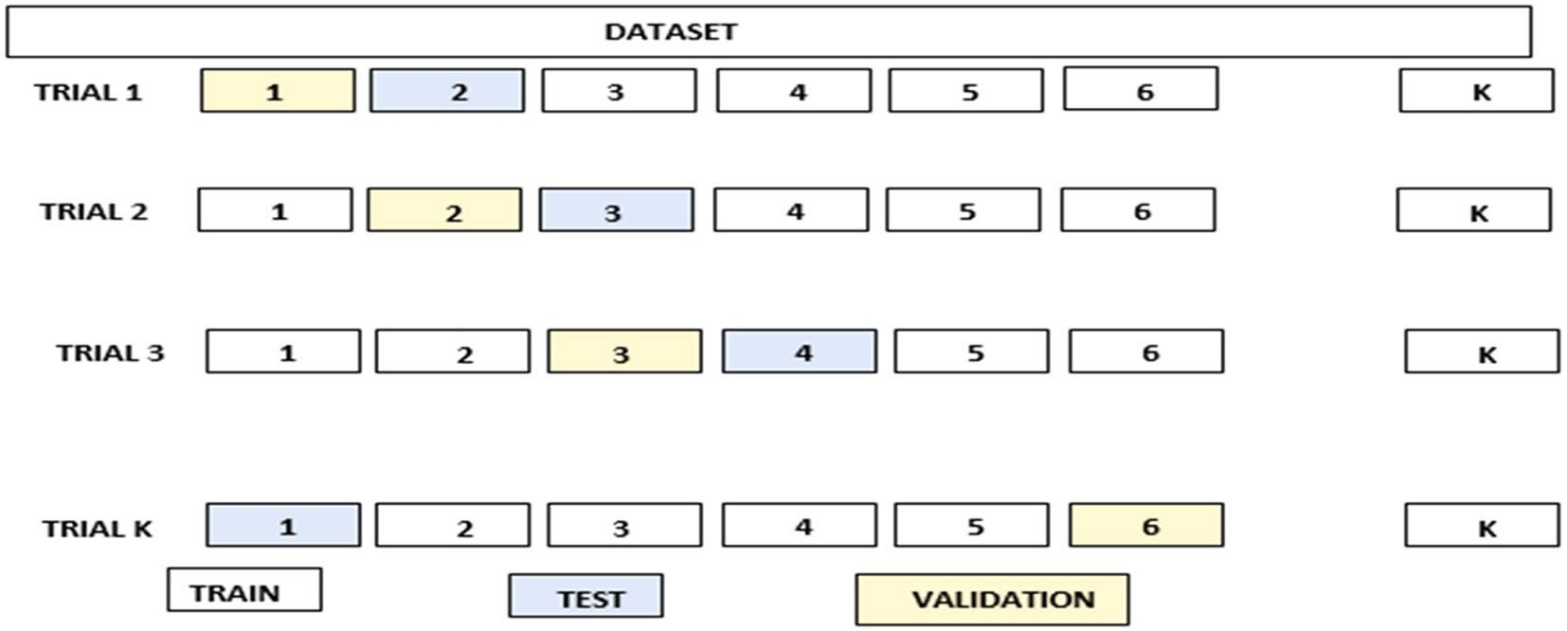
Visual representation of k-fold cross-validation.

In this work, *k*-fold cross-validation has been performed. Here, *k* stands for the number of validations and *N* is the number of samples in the *x* dataset. Hence, cross-validation is like bootstrapping^79^. Ten-fold cross-validation is used in this work and hence 10 sets of data are used. A representation of the cross-validation is as given in Fig. 15.

### Classification kernels

The kernel function in a classifier is used to transform a nonlinear decision surface to a linear surface. The robustness of the classifier depends on the properly adjusted parameter in kernel functions^80^. Many kernel functions are developed as per the Hilbert–Schmidt theory and Mercer condition^81^. Construction of the basic kernel can be done by changing the parameters of the kernel with same input features or by changing the input features for each kernel with fixed kernel parameters or by changing the features and parameters simultaneously^82^. Six different kernels are considered here for research.

#### Dot kernel

Dot kernel is the most common kernel in the classifier. Dot kernel is the product of *x* and *y* inner variables. The dot kernel is defined in Eq. 27

27 $${\text{k}}\;\left( {{\text{x}},\;{\text{y}}} \right) = {\text{x}}*{\text{y}}$$

where *k* is the dot kernel and *x* and *y* are variables. The dot kernel is in vogue now, with regard to SVM^83^.

#### Radial kernel

The radial kernel is defined in Eq. (28)

28 $$k\left(x,y\right)=\mathrm{exp}(-\gamma \sum_{j=1}^{p}{{(x}_{ij}{-y}_{ij})}^{2})$$

$\gamma$—tuning parameter that accounts for the decision boundary's smoothness and governs the model's variance.

*K*—radial kernel, *x* and *y* are variables.

This kernel is used when there is no knowledge of the data beforehand^84^

#### Polynomial kernel

The polynomial kernel is used to match the data with the normalized training data^85^. The polynomial kernel is defined in Eq. (29).

29 $$k\;\left( {x,y} \right) = \left( {x*y + {1}} \right)$$

where *p* is the degree of the polynomial, *x* and *y* are variables, and *k* is the polynomial kernel.

#### Multiquadric kernel

Multiquadratic is a classic example of non-positive definite kernel^86^.

The multiquadric kernel is defined in Eq. (30)

30 $$k\;\left( {x,y} \right) = \left( {\left| {\left| {x - y} \right|} \right|{2} + {\text{c}}^{{2}} } \right)\;0.{5}$$

where c is a constant,* K* is the multiquadric kernel, and *x* and *y* are variables.

#### Epanechnikov kernel

The Epanechnikov kernel can be defined in Eq. (31).

31 $$k\left(u\right)=\frac{3}{4}\left({1-u}^{2}\right)\mathrm{for }\left|u\right|\le 1$$

where* k* is the Epanechnikov kernel and *u* is variable^87^.

#### ANOVA kernel

ANOVA kernel is defined in Eq. (32).

32 $$k\left(x,y\right)=\sum_{k=1}^{n}{exp(-\sigma ({x}^{k}-{y}^{k})}^{2} )$$

where *k* is the ANOVA kernel, *x*, *y*, and *k* are variables, *n* is the number of images.

ANOVA is known to perform better in multidimensional problems^88^.

### Sampling

Sampling is used for analyzing the image pixels and modifying the pixels. The sampling rate identifies the spatial resolution of the digital image. A sample image is taken, and its magnitude is represented in a digital value which is considered as the sample. The sampling techniques used in the research are stratified sampling, linear sampling, shuffle sampling, and automatic sampling.

#### Stratified sampling

Stratified sampling is a type of sampling where the pixels are divided into smaller groups known as strata. Then a sample is taken randomly from each group for the analysis. The entire image will be taken, and missing important features will be less.

#### Linear sampling

Linear sampling is used to solve the problem of inverse diffraction in acoustics. The linear sampling method (LSM) is a basic and reliable way of image shaping. It shapes the unknown targets through a linear inverse problem solution.

#### Shuffle sampling

The shuffle sampling takes place by shuffling the pixels randomly and the pattern will be generated with good distribution in each dimension. This is also called a padding approach. If this shuffling was not performed, the values of the sample dimensions would be associated in such a way, which leads to image errors.

#### Automatic sampling

This sampling converts the images into a digitalized format. The continuous data is sent for digitalizing the image. Automatic sampling is the process of cataloguing the co-ordinate values automatically. Digitizer's automatic sampling frequency defines the digitized image's spatial resolution.

## Results

### Results of classification of uncalibrated images using SVM

The linear sampling for all the kernels of LSB replacement, LSB matching, and F5 in Table 3 give a classification of 51.92%. Most of the classification percentage for LSB replacement and LSB matching has the range between 75 and 84%. The radial and Epanechnikov kernels have the classification percentage of 75% and 77% for all sampling. The dot, polynomial, multiquadric, and ANOVA have a classification percentage of 81% to 84%. The ANOVA, Epanechnikov, and polynomial kernels have the classification from 40 to 48% with shuffled, stratified, and automatic kernels. The dot and multiquadric kernels have classification between 50 and 61%. The F5 algorithm has classification percentage ranging from 80 to 97%.

**Table 3:** Classification of uncalibrated images using SVM for random %.

	Linear	Shuffle	Stratified	Automatic
LSB replacement				
Dot	51.92	81.01	81.01	81.01
Radial	51.92	75.36	76.44	76.44
Polynomial	51.92	81.01	80.65	80.65
Multiquadric	51.92	81.01	80.77	80.77
Epanechnikov	51.92	76.44	77.28	77.28
ANOVA	51.92	83.65	**84.5**	**84.5**
LSB matching				
Dot	51.92	81.01	81.01	81.01
Radial	51.92	75.36	76.44	76.44
Polynomial	51.92	81.01	80.53	80.53
Multiquadric	51.92	81.01	80.77	80.77
Epanechnikov	51.92	76.44	77.28	77.28
ANOVA	51.92	**84.86**	84.5	84.5
PVD				
Dot	37.5	**61.25**	55.31	55.31
Radial	37.5	25.65	28.12	28.12
Polynomial	37.5	48.75	43.44	43.44
Multiquadric	37.5	50	50	50
Epanechnikov	37.5	41.56	40.62	40.62
ANOVA	37.5	47.19	48.12	48.12
F5				
Dot	51.92	96.51	95.43	95.43
Radial	51.92	81.01	80.77	80.77
Polynomial	51.92	91.59	91.47	91.47
Multiquadric	51.92	81.01	80.77	80.77
Epanechnikov	51.92	81.01	80.77	80.77
ANOVA	51.92	**97.6**	96.75	96.75

Significant values are in bold.

**Graph 1:**
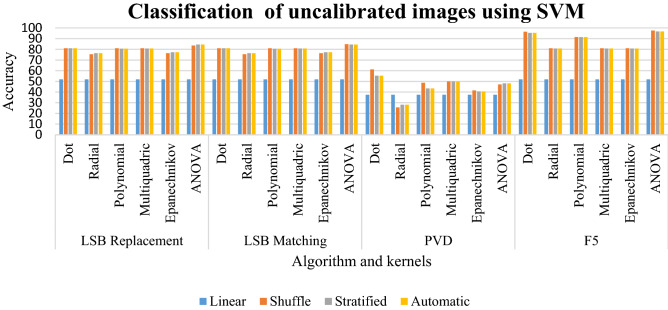
Result of classification of uncalibrated images using SVM for random %.

For the given specification, the linear sampling for LSB replacement, LSB matching, and F5 has a classification of 51.92. The other samplings for all the kernels have a classification ranging between 75 and 97%. LSB replacement has a better sampling of 84.5 for ANOVA in its stratified and automatic sampling. LSB matching has a better sampling of 84.86%. In PVD, better classification is given by dot kernel in its shuffled sampling. The F5 has a better classification rate in its ANOVA kernel with shuffled sampling. This is clearly depicted in Graph 1.

### Results of classification of calibrated images using SVM

**Graph 2:**
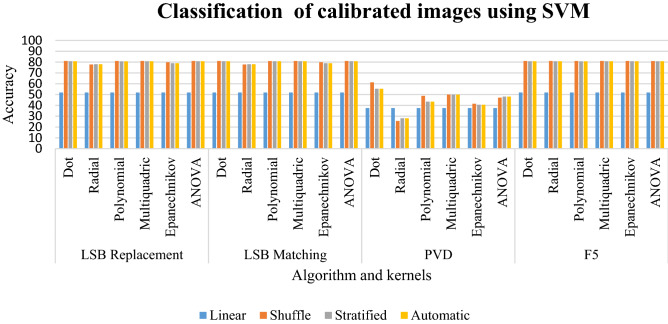
Result of classification of calibrated images using SVM for random %.

The linear sampling in Table 4 has classification of 51.92 for LSB replacement, LSB matching, and F5. In LSB replacement, the classification percentage is from 77 to 81 for shuffled, stratified, and ANOVA sampling. For LSB matching, the classification percentage is from 77 to 81 for all sampling. The ANOVA, Epanechnikov, and polynomial give a percentage between 40 and 48%. The dot kernel gives a percentage of 55% to 61% for the above sampling. For the F5 algorithm, all sampling has the percentage between 80 and 81 for all kernels. The linear sampling gives a percentage of 51.92 for all kernels. The PVD kernel give a low classification of 37.5 for linear sampling. The other steganographic schemes give a moderate sampling of 51.92. All steganographic schemes with different kernels give a classification percentage between 77 and 81% except the PVD steganographic scheme. LSB replacement has a better classification of 81.01 with shuffled sampling for dot, polynomial, multiquadric, and ANOVA kernels. For the PVD scheme, a good classification of 61.25 is given by the dot kernel in shuffled sampling. However, for the F5 steganographic algorithm, better classification is given by all kernels in shuffled sampling except ANOVA kernel. This is clearly depicted in Graph 2.

**Table 4:** Classification of calibrated images using SVM for random %.

	Linear	Shuffle	Stratified	Automatic
LSB replacement				
Dot	51.92	**81.01**	80.77	80.77
Radial	51.92	77.76	78	78
Polynomial	51.92	**81.01**	80.65	80.65
Multiquadric	51.92	**81.01**	80.77	80.77
Epanechnikov	51.92	79.81	78.97	78.97
ANOVA	51.92	**81.01**	80.77	80.77
LSB matching				
Dot	51.92	**81.01**	80.77	80.77
Radial	51.92	77.76	78	78
Polynomial	51.92	80.89	80.77	80.77
Multiquadric	51.92	**81.01**	80.77	80.77
Epanechnikov	51.92	79.81	78.97	78.97
ANOVA	51.92	**81.01**	80.77	80.77
PVD				
Dot	37.5	**61.25**	55.31	55.31
Radial	37.5	25.62	28.12	28.12
Polynomial	37.5	48.75	43.44	43.44
Multiquadric	37.5	50	50	50
Epanechnikov	37.5	41.56	40.62	40.62
ANOVA	37.5	47.19	48.12	48.12
F5				
Dot	51.92	**81.01**	80.77	80.77
Radial	51.92	**81.01**	80.77	80.77
Polynomial	51.92	**81.01**	80.65	80.65
Multiquadric	51.92	**81.01**	80.77	80.77
Epanechnikov	51.92	**81.01**	80.77	80.77
ANOVA	51.92	80.89	80.77	80.77

Significant values are in bold.

### Results of classification of uncalibrated images using SVM-PSO

In LSB replacement, the ANOVA, and radial kernels in linear sampling in Table 5 have a percentage ranging from 33 to 36. The other kernels in linear sampling have a percentage from 50 to 51. The ANOVA kernel for the above sampling has the classification between 66 and 68%. The Epanechnikov, radial, and polynomial have a percentage between 75 and 81. The same pattern is followed by LSB matching. The ANOVA has a classification of 99.8%. The Epanechnikov kernel has classification between 38 and 40%. The radial and multiquadric kernels have classification between 44 and 54%. The polynomial kernel gives the classification between 53 and 69%.

**Table 5:** Classification of uncalibrated images using SVM-PSO for random %.

	Linear	Shuffle	Stratified	Automatic
LSB replacement				
Dot	50.96	37.86	38.94	38.94
Radial	36.06	75.36	73.8	73.8
Polynomial	50.36	**81.01**	80.77	80.77
Multiquadric	50.96	30.05	30.53	30.53
Epanechnikov	51.92	78.49	78.37	78.37
ANOVA	33.65	66.59	68.75	68.75
LSB matching				
Dot	50.96	37.86	38.94	38.94
Radial	36.06	75.24	73.68	73.68
Polynomial	50.36	**81.01**	80.77	80.77
Multiquadric	50.96	30.05	30.53	30.53
Epanechnikov	51.92	78.49	78.37	78.37
ANOVA	33.65	66.59	68.75	68.75
PVD				
Dot	37.5	64.38	50	50
Radial	56.56	44.06	48.44	48.44
Polynomial	47.81	53.12	69.69	69.69
Multiquadric	55.62	47.19	54.69	54.69
Epanechnikov	37.81	38.75	40.94	40.94
ANOVA	**99.8**	76.56	74.38	74.38
F5				
Dot	51.2	37.86	38.94	38.94
Radial	38.58	77.76	77.52	77.52
Polynomial	50.24	**81.01**	80.77	80.77
Multiquadric	51.44	30.65	31.25	31.25
Epanechnikov	51.92	**81.01**	80.53	80.53
ANOVA	43.03	68.15	72.96	72.96

Significant values are in bold.

**Graph 3:**
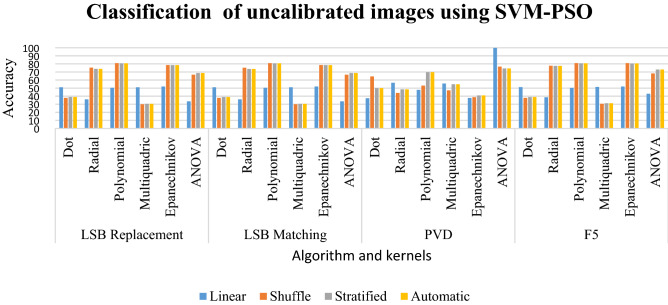
Result of classification of uncalibrated images using SVM-PSO for random %.

For LSB replacement, most of the classification is between 73 and 81%. The better classification is given by the polynomial in its shuffled kernel. For LSB matching, the pattern is followed with better classification given by the polynomial for its shuffled sampling. The PVD scheme has a very good classification with the ANOVA giving a classification of 99.8 in its linear sampling. The F5 scheme also gives good classification between 68 and 81%. The better classification is given by polynomial and Epanechnikov kernels in shuffled sampling. This is clearly depicted in Graph 3.

### Results of classification of calibrated images using SVM-PSO

The linear sampling in Table 6 has classification ranging from 37 to 53 for all steganographic algorithms. The radial kernel has the percentage between 61 and 65%. The ANOVA and Epanechnikov kernels have classification ranging from 69 to 77%. The polynomial kernel gives a classification between 80 and 81%. LSB matching follows the same pattern as LSB replacement. For the F5, the classification percentage of dot and multiquadric is from 29 to 38% for shuffled, stratified, and automatic sampling. The radial and ANOVA kernels have the classification between 63 to 70%. The Epanechnikov kernel has a classification of 80%.

**Table 6:** Classification of calibrated images using SVM-PSO for random %.

	Linear	Shuffle	Stratified	Automatic
LSB replacement				
Dot	51.68	36.42	36.9	36.9
Radial	45.91	65.02	61.9	61.9
Polynomial	50	**81.01**	80.77	80.77
Multiquadric	51.08	29.57	29.69	29.69
Epanechnikov	51.92	76.8	77.52	77.52
ANOVA	49.64	70.19	69.11	69.11
LSB matching				
Dot	51.68	36.42	36.9	36.9
Radial	45.91	65.14	62.26	62.26
Polynomial	50	**81.01**	80.77	80.77
Multiquadric	51.08	29.57	29.69	29.69
Epanechnikov	51.92	76.92	77.52	77.52
ANOVA	49.76	70.19	69.11	69.11
PVD				
Dot	44.38	50	50	50
Radial	53.12	42.19	37.5	37.5
Polynomial	50.62	49.06	48.12	48.12
Multiquadric	**54.69**	50.62	52.12	52.12
Epanechnikov	37.19	39.38	37.5	37.5
ANOVA	37.5	45.94	45.94	45.94
F5				
Dot	53	37.38	38.1	38.1
Radial	48.56	67.31	63.22	63.22
Polynomial	51.32	**81.01**	80.77	80.77
Multiquadric	51.8	31.01	29.69	29.69
Epanechnikov	51.92	80.05	80.17	80.17
ANOVA	49.28	70.31	68.75	68.75

Significant values are in bold.

**Graph 4:**
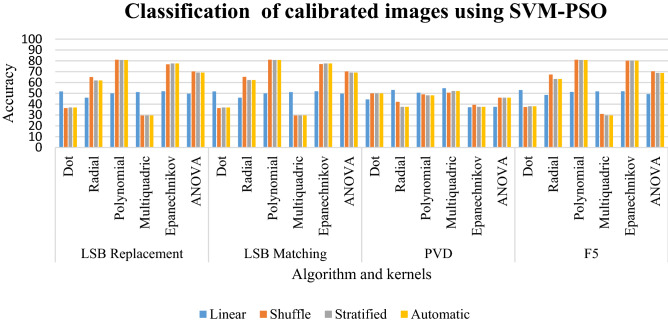
Result of classification of calibrated images using SVM-PSO for random%.

For LSB replacement, the dot and multiquadric have a low classification percentage between 29 and 36. The Epanechnikov kernel and ANOVA give a good classification. A better classification is given by the polynomial kernel in shuffled sampling. LSB matching follows the same pattern as LSB replacement. The PVD scheme has a comparatively reduced classification rate. The good classification rate in this scheme is displayed by the multiquadric kernel in linear sampling. The F5 scheme follows nearly the same pattern as LSB replacement and LSB matching. The good classification is given by the polynomial kernel in shuffled sampling. This is clearly depicted in Graph 4.

### Results of classification of uncalibrated images with cross-validation using SVM

In LSB replacement in Table 7, the dot and multiquadric kernels give a classification for all samplings in the range of 31% to 39%. The radial kernel has the classification between 54 to 57%. The ANOVA kernel exhibits a classification ranging from 64 to 85%. For LSB matching, most of the kernels have a classification between 73 and 80% except ANOVA which has a classification of 64%. The PVD scheme gives the classification of Epanechnikov and radial to be in the range of 25 to 29 for all sampling. The multiquadric kernel has the classification between 46 and 50%. The F5 has all the classification of all kernels to be more than 80%.

**Table 7:** Classification of uncalibrated images with cross-validation using SVM for random %.

	Linear	Shuffle	Stratified	Automatic
LSB replacement				
Dot	38.66	39.4	39.06	39.06
Radial	54.07	57.19	56.04	56.04
Polynomial	68.32	59.73	59.4	59.4
Multiquadric	33.08	31.46	31.55	31.55
Epanechnikov	75.19	76.04	76.05	76.05
ANOVA	64.46	64.65	**85.04**	**85.04**
LSB matching				
Dot	79.33	**80.95**	80.9	80.9
Radial	72.96	74.22	74.31	74.31
Polynomial	79.23	80.76	80.81	80.81
Multiquadric	80.77	80.76	80.76	80.76
Epanechnikov	73.85	75.32	75.37	75.37
ANOVA	64.45	64.65	64.45	64.45
PVD				
Dot	62.25	**74.75**	74.5	74.5
Radial	5.25	25.38	29	29
Polynomial	63.62	72.88	73	73
Multiquadric	10	46.25	50	50
Epanechnikov	5.12	26.5	26.38	26.38
ANOVA	56	74.12	74	74
F5				
Dot	87.45	**97.21**	97.16	97.16
Radial	80.77	80.76	80.76	80.76
Polynomial	85.67	91.87	91.77	91.77
Multiquadric	80.77	80.76	80.76	80.76
Epanechnikov	80.77	80.76	80.76	80.76
ANOVA	66.47	68.21	68.11	68.11

Significant values are in bold.

**Graph 5:**
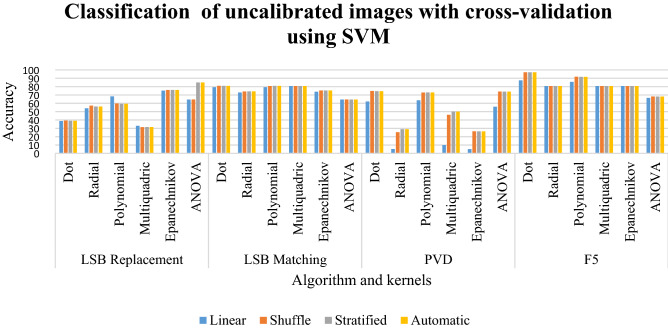
Result of classification of uncalibrated images with cross-validation using SVM for random %.

In LSB replacement, the dot and multiquadric kernels have a low classification rate. The radial and polynomial kernels give a moderate classification rate. The better classification is given by ANOVA in their stratified and automatic sampling. In LSB matching, most of the kernels and samplings give a good classification. The better classification is displayed by the dot kernel in shuffled sampling. In the F5 algorithm, most of the kernels and sampling have high classification percentages. However, better classification is done by the dot kernel in shuffled sampling. This is clearly depicted in Graph 5.

### Results of classification of calibrated images with cross-validation using SVM

In LSB replacement and LSB matching in Table 8, most of the kernels with different sampling has the classification percentage between 75 to 80%. But the ANOVA kernel has a classification between 65 and 66%. The polynomial kernel has a classification of 36% to 38% for shuffled, stratified, and automatic kernels. But the polynomial in linear sampling gives a classification of 52.5%. The multiquadric kernel has the classification between 46 and 50% for shuffled, stratified, and automatic sampling. The linear sampling has a classification of 73.25%. The F5 scheme has the classification between 77 to 80% for all kernels and sampling.

**Table 8:** Classification of calibrated images with cross-validation using SVM for random %.

	Linear	Shuffle	Stratified	Automatic
LSB replacement				
Dot	75.67	80.76	80.76	80.76
Radial	76.59	77.78	77.87	77.87
Polynomial	77.64	80.76	80.76	80.76
Multiquadric	**80.77**	80.76	80.76	80.76
Epanechnikov	77.79	78.5	78.55	78.55
ANOVA	65.85	66.43	66.47	66.47
LSB matching				
Dot	75.48	80.76	80.76	80.76
Radial	76.59	77.78	77.87	77.87
Polynomial	77.69	80.71	80.76	80.76
Multiquadric	**80.77**	80.76	80.76	80.76
Epanechnikov	77.79	78.5	78.55	78.55
ANOVA	65.85	66.47	66.48	66.48
PVD				
Dot	13	60.38	61.25	61.25
Radial	27.13	26.38	24	24
Polynomial	52.5	38.62	36.88	36.88
Multiquadric	**73.25**	46.25	50	50
Epanechnikov	6.75	33	33.38	33.38
ANOVA	16.25	42.64	41.62	41.62
F5				
Dot	77.55	80.76	80.76	80.76
Radial	**80.77**	80.76	80.76	80.76
Polynomial	77.36	80.71	80.71	80.71
Multiquadric	**80.77**	80.76	80.76	80.76
Epanechnikov	**80.77**	80.76	80.76	80.76
ANOVA	65.04	66.33	80.71	80.71

Significant values are in bold.

**Graph 6:**
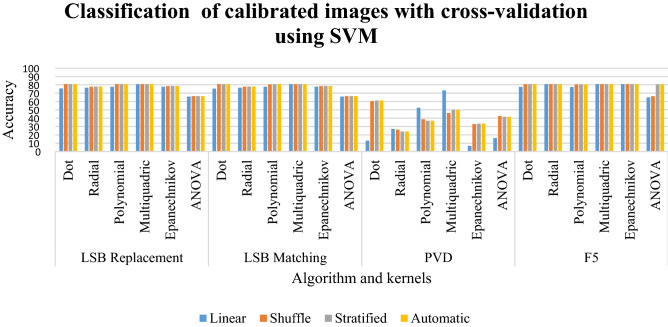
Result of classification of calibrated images with cross-validation using SVM for random %.

LSB replacement and LSB matching have most of the classification for all kernels and sampling to be between 75 and 80%. But the ANOVA kernel has a lesser classification between 65 and 66%. The better classification of LSB replacement and LSB matching is given by the multiquadric in linear sampling. For PVD steganographic scheme, the classification is a bit lower. The dot, Epanechnikov, and ANOVA kernel have very low classification for linear sampling. The better sampling is given by the multiquadric kernel for linear sampling. For the F5 steganographic scheme, almost all kernels and sampling give a good classification rate, but a better classification is given by radial, multiquadric, and Epanechnikov kernels in linear sampling. This is clearly depicted in Graph 6.

### Results of classification of uncalibrated images with cross-validation using SVM-PSO

In LSB replacement and LSB matching in Table 9, the dot kernel gives an accuracy between 38 and 39%. The multiquadric kernel has the classification between 30 and 32%. The radial kernel has a classification of 53% to 56%. But the ANOVA kernel has a classification of 64%. The Epanechnikov kernel gives an accuracy between 74 and 76%. The linear sampling has a classification of 72.75%. The F5 scheme has the classification between 57 and 68% for all radial and Epanechnikov kernels. The dot and multiquadric kernels have a classification between 31 and 39%. The Epanechnikov kernel has a classification of 80%.

**Table 9:** Classification of uncalibrated images with cross-validation using SVM-PSO for random %.

	Linear	Shuffle	Stratified	Automatic
LSB replacement				
Dot	38.86	39.16	39.01	39.01
Radial	53.78	56.9	56.85	56.85
Polynomial	**88.85**	63.62	59.4	59.4
Multiquadric	32.75	30.83	31.55	31.55
Epanechnikov	74.81	76.53	74.65	74.65
ANOVA	64.45	64.65	64.45	64.45
LSB matching				
Dot	38.86	39.11	39.01	39.01
Radial	53.88	56.9	55.99	55.99
Polynomial	**88.85**	63.52	59.32	59.32
Multiquadric	32.75	30.78	31.55	31.55
Epanechnikov	74.81	76.62	76.05	76.05
ANOVA	64.46	64.65	64.45	64.45
PVD				
Dot	22	49.12	50.62	50.62
Radial	44	41.37	40.25	40.25
Polynomial	26.88	56.75	53.37	53.37
Multiquadric	72.75	49.62	49.75	49.75
Epanechnikov	7.25	34.25	34.88	34.88
ANOVA	73	**74.13**	74	74
F5				
Dot	39.1	39.59	39.39	39.39
Radial	57.92	60.89	60.9	60.9
Polynomial	**86.54**	55.35	48.25	48.25
Multiquadric	33.03	31.6	31.89	31.89
Epanechnikov	80.14	80.85	80.47	80.47
ANOVA	66.58	68.21	68.11	68.11

Significant values are in bold.

**Graph 7:**
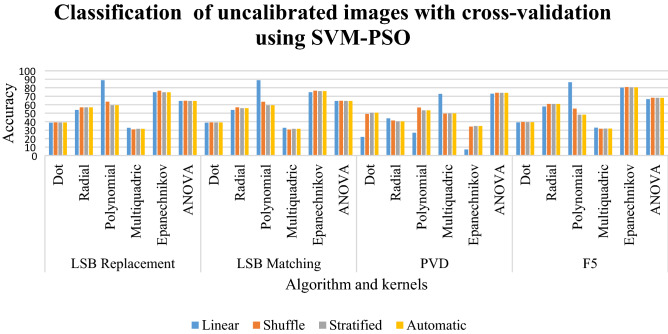
Result of classification of uncalibrated images with cross-validation using SVM-PSO for random %.

LSB replacement and LSB matching has low classification rates on dot and multiquadric sampling. A good classification is exhibited by the Epanechnikov kernel for all sampling. However, the classification is a bit lower for the ANOVA kernel. The better classification is given by the polynomial kernel in linear sampling. The classification in general for PVD scheme is lower. But the ANOVA displayed a good classification rate. The F5 algorithm follows the same pattern as LSB replacement and LSB matching, with Epanechnikov giving good classification results. The better classification is exhibited by the polynomial kernel in linear sampling. This is clearly depicted in Graph 7.

### Results of classification of calibrated images with cross-validation using SVM-PSO

In LSB replacement in Table 10, the dot and radial kernels have a classification between 29 and 37%. The radial kernel has a classification between 55 and 57%. The polynomial and Epanechnikov kernels have a classification between 74 and 75%. The ANOVA kernel has the classification between 65 and 66%. The ANOVA kernel has classification between 41 and 42%. The multiquadric kernel has classification between 46 and 50% for all sampling. The dot kernel has the classification between 60 and 61 in the above samplings. However, for F5, the classification is between 65 and 66%.

**Table 10:** Classification of calibrated images with cross-validation using SVM-PSO for random %.

	Linear	Shuffle	Stratified	Automatic
LSB replacement				
Dot	36.5	37.14	37.23	37.23
Radial	55.08	57.77	57.72	57.72
Polynomial	74.28	75.4	**75.57**	**75.57**
Multiquadric	30.34	29.58	30.26	30.26
Epanechnikov	74.66	75.18	75.23	75.23
ANOVA	65.85	66.09	66.48	66.48
LSB matching				
Dot	36.5	**80.76**	**80.76**	**80.76**
Radial	55.32	57.87	77.87	77.87
Polynomial	77.166	75.4	**80.76**	**80.76**
Multiquadric	31.21	29.58	30.26	30.26
Epanechnikov	73.65	74.99	75.28	75.28
ANOVA	65.85	66.47	66.48	66.48
PVD				
Dot	12.5	60.38	**61.25**	**61.25**
Radial	1.25	26.38	24	24
Polynomial	5.88	38.62	36.88	36.88
Multiquadric	10	46.25	50	50
Epanechnikov	7.62	33	33.38	33.38
ANOVA	6.38	42.12	41	41
F5				
Dot	77.55	80.76	80.76	80.76
Radial	**80.77**	80.76	80.76	80.76
Polynomial	77.36	80.71	80.71	80.71
Multiquadric	**80.77**	80.76	80.76	80.76
Epanechnikov	**80.77**	80.76	80.76	80.76
ANOVA	65.04	66.33	65.61	65.61

Significant values are in bold.

**Graph 8:**
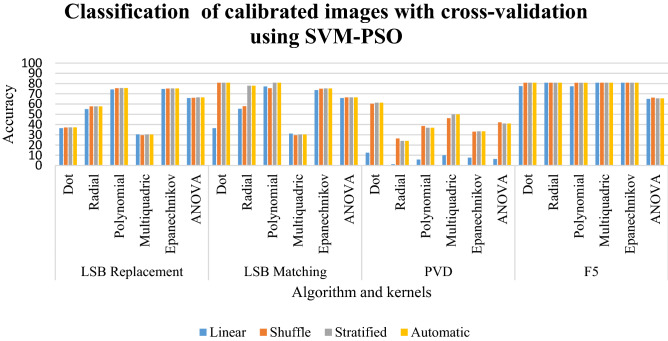
Result of classification of calibrated images with cross-validation using SVM-PSO for random %.

For LSB replacement, the dot and multiquadric kernels have low classification. The ANOVA has a moderate classification. A higher classification is given by Epanechnikov and polynomial kernels. For LSB matching, the multiquadric has low classification. The other classification has moderate to good classification rate. The better classification is given by the polynomial kernel in its stratified and automatic sampling. In the F5 algorithm, most classification give values of more than 77%. The better classification is given by linear sampling with dot, multiquadric, and Epanechnikov kernels. This is clearly depicted in Graph 8.

## Conclusion

In this research, the feature based steganalysis of uncalibrated and calibrated images with random embedding percentage is considered. Standard datasets available publicly for the research such as INRIA holiday dataset and UCID dataset. In which 1500 images of INRIA datasets had been used to train the classifier whereas 800 images from UCID image dataset had been used as test data. The concept of cross validation had been considered in this research. Principal Component Analysis had been used for dimensionality reduction. Various steganographic techniques both in spatial and transform domain had been considered for embedding the message. The steganographic algorithms are LSB substitution, LSB Matching and PVD in spatial domain and F5 in Discrete Cosine Transform domain. A comparative study of classification is done by means of SVM and its evolutionary counterpart SVM-PSO. Six different kernel functions and four sampling had been considered for the classification. Features are extracted from first order, second order and Markov.

The results clearly state that uncalibrated images classified with SVM-PSO give a better classification. The steganographic algorithm used is PVD. The kernel function incorporated is ANOVA with linear sampling. The other results give a better outcome mostly n shuffled sampling. F5 algorithm in transform domain give a better classification percentage than the other algorithms considered in the paper. The polynomial kernel function would also give a better classification percentage.

## Future scope

- The input data can be audio or video in different formats.
- Feature selection optimization like forward selection, backward elimination can be done.
- The first order, second order, DCT, and Markovian features are used for feature extraction in the research. Hybrid features can be considered as a future scope in the thesis.
- Cross-validation folds can be changed to check on the outcomes. Split validation, bootstrapping validation, or wrapper split validation can be used.
- Predictive analysis can be done using discriminant analysis or evolutionary logistic regression. Neural network or rule induction can also be used.
- Deep learning can be used for improved classification
- Steganalysis can be implemented in JPEG Medical Images, DICOM images and other image formats
- Extension of steganalysis to the field of cyber biosecurity.

## Data Availability

All datasets analyzed for the research can be publicly available in https://lear.inrialpes.fr/~jegou/data.php#holidays, https://qualinet.github.io/databases/image/uncompressed_colour_image_database_ucid/.
